# Prevention of lipid loss from hair by surface and internal modification

**DOI:** 10.1038/s41598-019-46370-x

**Published:** 2019-07-08

**Authors:** Sang-Hun Song, Jong Hyun Lim, Seong Kil Son, Julia Choi, Nae-Gyu Kang, Sang-Min Lee

**Affiliations:** LG Science Park in Magok, LG Household & Health Care, Gangseo-gu, Seoul, 07795 Republic of Korea

**Keywords:** Tissues, Preventive medicine

## Abstract

Surfactants during routine washing have a tremendous effect on lipid loss from hair. This study aims to understand the loss of lipids from hair upon contact with surfactants and develop a way to prevent the lipid loss. The change in lipid levels depends on the relative hydrophobicity of the lipid. We herein propose that the change in lipid levels can be protected by two modifications. In the case of fatty acids and cholesterol (group A), the concentration difference between virgin hair versus surface modified hair with highly charged polymer was 22 to 32% higher after washing with surfactants while the loss of squalene and wax esters (group B) in response to surfactants still occurred even after the surface modification. In the hair treated by internal modification with the carbodiimide reaction, 52.0 to 81.3% more lipids in group B were prevented than in the untreated hair. Finally, different types of lipids were successfully protected by surface and internal modifications from the surfactant treatment. This study will be the basis for understanding the mechanisms by which surfactants damage the lipid barrier of tissues including hair and for establishing strategies to defend the barrier.

## Introduction

Almost all organisms and tissues utilize a lipid barrier for protection from the external environment^1^. In the case of hair, a laminated structure of lipid molecules such as fatty acids, ceramides, glycolipids, and cholesterols plays a role in the protection^2^. This lipid barrier is essential for preventing the penetration of foreign matter and the loss of internal moisture^2–4^. However, due to routine washing with surfactants, these barriers for skin are constantly damaged and eventually lose their functions^5^.

The lipids in hair shaft are formed in the follicle^2^. Sebum secretion onto the surface is, more or less, a continuous process, so sebaceous lipids that are lost will be replenished. However, much of the internal lipid including ceramides, cholesterol, fatty acids and cholesterol sulfate are not derived from sebum. Since sebum lipids are not the sole source for the hair lipids, the lipids in hair shaft must be restored incompletely once they are lost. Hence, it is required to accurately characterize lipid loss in hair and to establish a solution that can fundamentally prevent loss. Unfortunately, due to different hydrophobicity of lipids presented in the hair, it is difficult to determine the effect of surfactants on lipid. Moreover, a complex structure of cell membrane complex (CMC) makes it more difficult to interpret the location at which the surfactants act.

Recently, outstanding researches on hair that deal with fibrous growth and new conditioning polymers using atomic force microscope (AFM) and chromatography have recently been actively reported^6–8^. In order to identify the characteristics of the loss of hair lipids, a detailed design of the analytical method is required. Characterization of hair lipids is also one of the most important topics to be systematically addressed^9–15^. However, a few studies have been conducted to directly characterize the loss of lipids^16^. Thus, the optimization of efficient techniques for simultaneously analyzing the amount of various lipids was required to characterize the loss of lipids from hair.

We have optimized the way to simultaneously quantify four individual fatty acids, cholesterol, squalene and three individual wax esters contained in hair. Also, the characteristics of lipid loss by surfactants could be identified. This article shows that the hydrophobicity of lipids distinguishes the mechanisms of lipid loss in response to surfactant treatment. This study is one of interfacial system models explaining the cause of lipid loss from hair and proposes effective solutions to prevent the lipid loss using polymeric materials that modify the exterior and interior of hairs.

## Results

### Identification and analysis of hair lipids

First, a method for extracting hair lipids had to be optimized. The best efficiency of extraction was obtained by sonication with 250 mg hair in a series of 200 mL portions of chloroform:methanol (2:1, 1:1, and 1:2) for 3 h each, followed by 18:9:1 chloroform:methanol:water for 9 h. Effective equipment was then chosen to simultaneously analyze the lipids extracted from hair. A comparison of the individual lipid concentrations determined by the gas chromatography/mass spectrometry (GC/MS), GC-flame ionization detector (GC/FID), and high-performance liquid chromatography (HPLC) is given in Supplementary Table 1. Among them, GC/MS was the most suitable for simultaneously measuring fatty acids, hydrocarbons, squalene, cholesterol and wax esters. Finally, GC/MS was employed to analyze the concentration of lipids. A detailed description of the methods for extraction is included in supporting materials and methods (Supplementary Figs 1–6, and Supplementary Tables 1–3).

Next, we determined the types of lipid to analyze. Hair lipids are classified into eight conventional groups: fatty acids, wax esters, squalene, cholesterol, 18-methyl eicosanoic acid (18-MEA), ceramides, hydrocarbons, and triglycerides^2^. Since 68.5% of total fatty acids are myristic acid (C14), palmitic acid (C16) and stearic acid (C18)^2^, these lipids were analyzed in this study. The content of wax esters is approximately 20% of the total lipid content^2^. Most wax esters consist of three forms, C14, C16 and C18^15^. Thus, myristyl palmitate (C14-C16), palmityl palmitate (C16-C16) and stearyl palmitate (C16-C18) were employed as representative wax esters. Squalene and cholesterol are also important lipids in hair, and the concentrations of them were determined. Ceramides, hydrocarbons, and triglycerides were not considered because their concentrations were too low (Supplementary Figs 1, 4). In conclusion, it was decided to analyze C14, C16, C18, C18=1, C14-C16, C16-C16, C16-C18, squalene, and cholesterol in this study (Supplementary Fig. 5).

### Lipid loss by washing with surfactant

**Figure 1:**
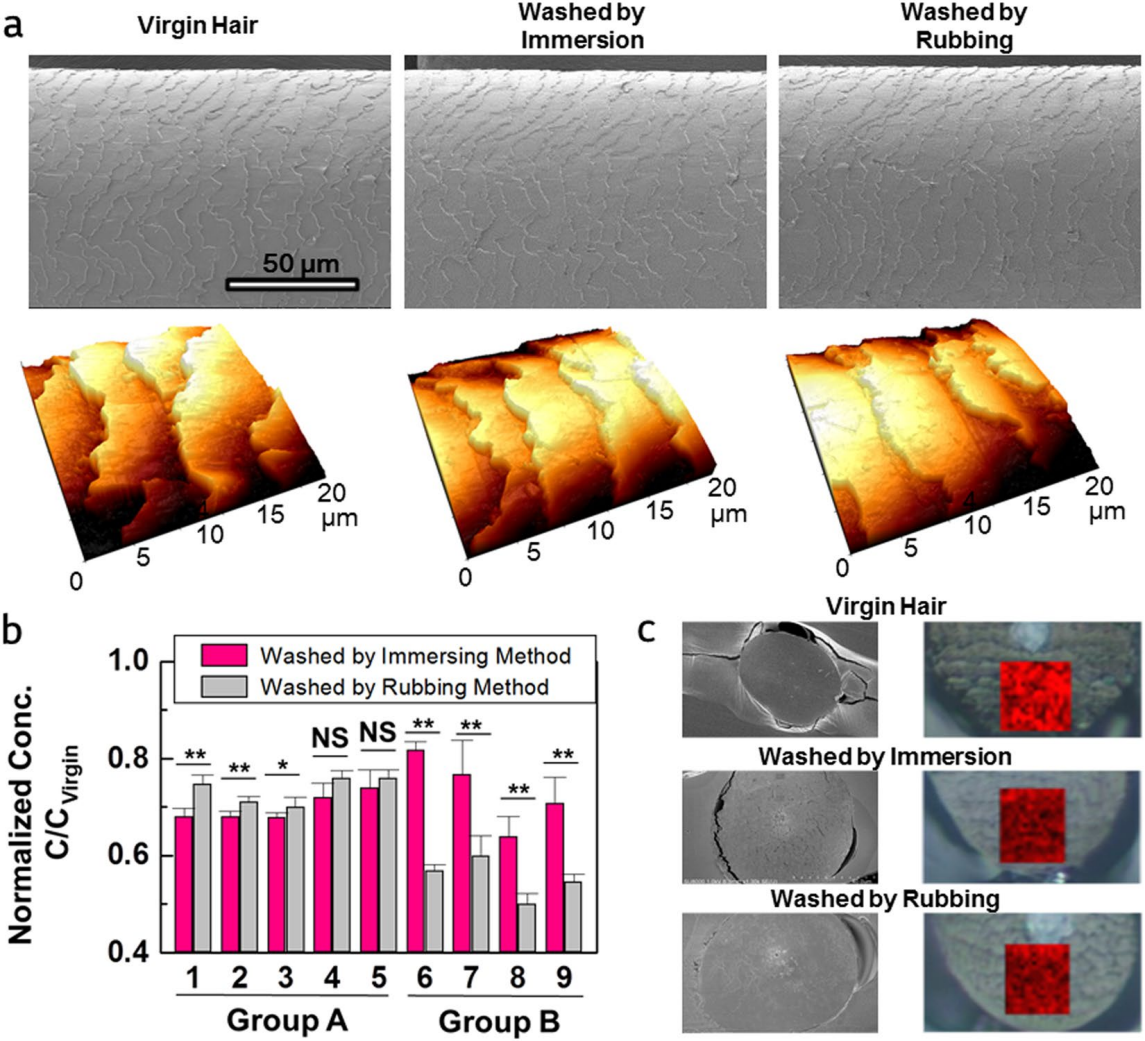
Comparison of virgin hair, hair washed by the immersion method, and hair washed by the rubbing method. (**a**) SEM and AFM images of washed hairs. (**b**) Noncovalently bound hair lipid composition after washing with an SLES solution. The concentration of lipids extracted from washed hair was divided by that from virgin hair. The error bar amplitude indicates standard deviation. The lines above the bar graph denote a significant difference (n = 6) calculated using Student’s t-test. NS – not significant, *P < 0.05, and **P < 0.01. Graphs for the lipids in group A: 1. Myristic acid; 2. Palmitic acid; 3. Stearic acid; 4. Oleic acid; 5. Cholesterol. Graphs for the lipids in group B: 6. Squalene; 7. Myristyl palmitate; 8. Palmityl palmitate; 9. Stearyl palmitate. (**c**) Spatial verification of lipid loss. SEM images of the ultramicrotomed hair coated with Pt (left), optical microscopy images and the corresponding *in situ* Raman image (right). A bright color means a high Raman intensity, i.e., a high density of wax esters.

To study the behavior of lipids with surfactants, two kinds of hair swatches were tested with sodium laureth sulfate (SLES) by the rubbing method and the immersion method. First, it was determined whether the rubbing method caused physical damage to the cuticle layer on the surface of the hair. The damage, such as cuticle peeling, can be identified by monitoring the morphology using a scanning electron microscope (SEM) and an AFM. Comparing virgin and washed hairs revealed no specific reduction of the cuticle density, as shown in Fig. 1a. In addition, the amount of 18-MEA located at the outermost side of the cuticle and covalently bound to underlying proteins through a thioester linkage did not show a significant difference between before and after washing (Supplementary Fig. 7). These results mean that the loss of lipids by washing was not derived by physical damage of cuticles layers.

Figure 1b shows normalized concentrations of lipids when hair was immersed for 10 min and rubbed for 10 cycles. The total time required for 10 cycles of rubbing was 10 min. Therefore, the contact time between the hair and the surfactant was the same in both methods. Since the hair was pre-rinsed to remove peripheral lipids (Supplementary Table 4), all lipids found by GC/MS were originally embedded in the hair. An interesting pattern was observed between two groups: higher amounts of C14 and C16 were lost when the hair was immersed than when the hair was rubbed. Although there was no significant difference between the residual concentrations of oleic acid and cholesterol after the rubbing and immersion treatments, the same pattern in which the amount lost by immersion was greater than that by rubbing was observed. Conversely, squalene and wax esters exhibited less loss when the hair was immersed than when rubbed.

Raman measurements were then performed on the cross-sectioned hair to determine where the lipids were extracted. To determine the distribution of lipids, examination of the signal was focused on the ester band which is associated with the C=O vibration and occurs at 1740 cm^−1^ ^17^. This band provides a practical way to deduce the location of wax esters as well as lipids in the CMC^2^. Additionally, the ester band at 1740 cm^−1^ does not overlap with the bands from proteins. Hence, the band at 1740 cm^−1^ can be used as a representative band to indicate the presence of lipids. Before measuring the Raman spectra, we observed that the cross-section was flat enough to produce an accurate Raman signal using a SEM as shown in Supplementary Fig. 8. The result confirming the lipid loss by the established method is presented in Fig. 1c. In the Raman image, the amount of lipids was identified by the intensity of the red color. As shown in Fig. 1c, a high intensity was observed in virgin hair, and the intensity decreased after washing, which indicates lipid loss. An important finding from these images is that the lipid loss did not occur in a specific region but occurred with a uniform distribution.

### Surface modification of hair

To understand the mechanisms by which lipids are lost by contact with surfactants, surface modification to increase the surface potential was attempted. The surface potential was increased using highly charged cationic polymers, polyquaternium-10B (PQ10B), PQ10C and PQ10D. These polymers have 2.0%, 2.4% and 2.8% (w/w) nitrogen, respectively. These polymers used can be regarded as highly charged because typical cationic polymers have approximately 1.0% nitrogen (Supplementary Fig. 9). While the complexes of cationic polymer and anionic surfactant involve association of near-neutral inter-polymer complexes, dilution-induced coacervation proceeds^18,19^. Coacervation is a phase separation from the bulk solution formed by the cationic PQ polymers with anionic surfactant systems. The surface modification was controlled by the coacervation to enhance the adsorption of the PQ film.

**Figure 2:**
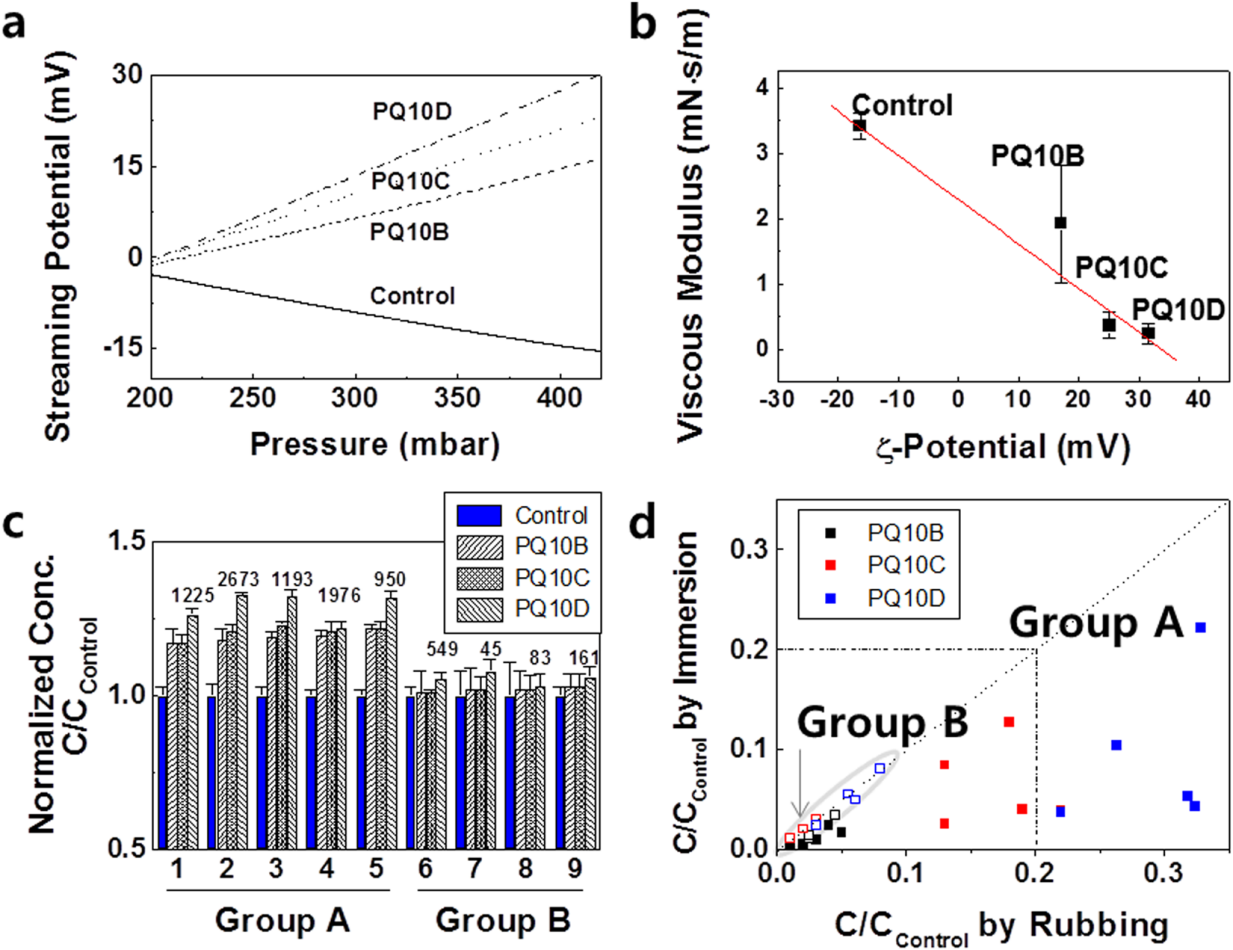
Effect of surface modification of hair on the properties of lipid loss in response to surfactant. (**a**) Streaming potential values of hairs treated with positively charged polymers (%N >2.0, w/w) and the control (%N .01.0, w/w) as a function of pressure. (**b**) Viscous modulus of polymers as a function of ζ-potential, obtained in (**a**), (**c**) The concentrations of the remaining lipids from the control and the surface-modified hair after being washed by rubbing 10 times. The normalized concentration was calculated based on the average concentration of the control. The numbers represent the actual concentrations (μg/g hair) of lipids extracted from PQ10D-treated hair. The numbers on the x-axis represent lipid described in Table 1. (**d**) Normalized concentrations from surface modification with rubbing wash and immersion extraction. Filled square: lipid in group A, Open mark: lipid in group B.

When the hair was washed with an SLES solution containing highly charged cationic polymers, the charge potential was effectively increased (Fig. 2a). The averaged ζ-potential was determined to be −16.3 mV in the case of the control hair which is treated with an SLES solution containing a typical cationic polymer having 1.0% nitrogen. However, PQ10B, PQ10C and PQ10D were found to provide appropriate cationic properties. The averaged ζ-potential was determined to be 17.1, 25.1, and 31.6 mV for the PQ10B, PQ10C and PQ10D, respectively. It is also determined that the addition of the cationic polymers to the SLES solution did not affect the lipid loss during the washing processes (Supplementary Table 5).

**Table 1:** Classification of lipids that were analyzed. The table shows average concentrations of lipids extracted from virgin hair.

Group	Species	C_Virgin_ (μg/g hair)
A	1. Myristic acid (C14)	1285 ± 34
	2. Palmitic acid (C16)	2763 ± 55
	3. Stearic acid (C18)	1274 ± 23
	4. Oleic acid (C18 . 1)	2096 ± 38
	5. Cholesterol	891 ± 17
B	6. Squalene	874 ± 17
	7. Myristyl palmitate (C14-C16)	60 ± 3
	8. Palmityl palmitate (C16-C16)	144 ± 5
	9. Stearyl palmitate (C16-C18)	243 ± 12

Viscous modulus was measured using pendant drop method in Fig. 2b. The viscous modulus decreased in order of control, PQ10B, PQ10C, and PQ10D. This result shows that the viscous modulus decreases as the ζ-potential decreased (namely, as the content of nitrogen increases).

After the surface was cationically modified, the pattern in which the lipid was lost from hair in response to surfactants was verified (Fig. 2c). Compared to that in the control group, in the surface-modified group with highly charged cationic polymers, the amount of the components of group A, such as fatty acids and cholesterol, remained 22 to 32% higher after washing with surfactants. In addition, the nitrogen content of the polymer was proportional to the inhibitory effect on lipid loss. These results imply that the loss of lipids in group A is highly dependent on the surface charge. Unlike the results for the components of group A, the surface modification did not significantly inhibit the loss of squalene and wax esters (group B).

The concentrations from surface modification were compared in rubbing wash and immersion extraction (Fig. 2d). In the case of lipids in group B, the difference of concentrations between the immersion and rubbing methods was similar. However, the normalized concentrations by rubbing are higher than those in immersion for lipids in group A. Regardless of the content of nitrogen in the polymer, the pattern in which more of these lipids was lost with the immersion method than with the rubbing method remained the same. This indicates that the rubbing method in the amphiphilic rubber extraction played the role of the diffusion on the surface.

### Internal modification of hair

The losses of squalene and wax esters were not significantly affected by surface modification. We hypothesized that these lipids were lost by penetration of the surfactant into the hair. To prove this hypothesis, hair was internally modified by filling with amine-coupling components. A reaction of carbodiimide with carboxylic acid of the hair increased local density for the inside of hair. The carbodiimide chemistry produces an unstable O-acylisourea intermediate^20^, and the intermediate is easily displaced by nucleophilic attack from amino groups. The primary amines consequently form new amide bonds with the initial carboxyl groups.

**Figure 3:**
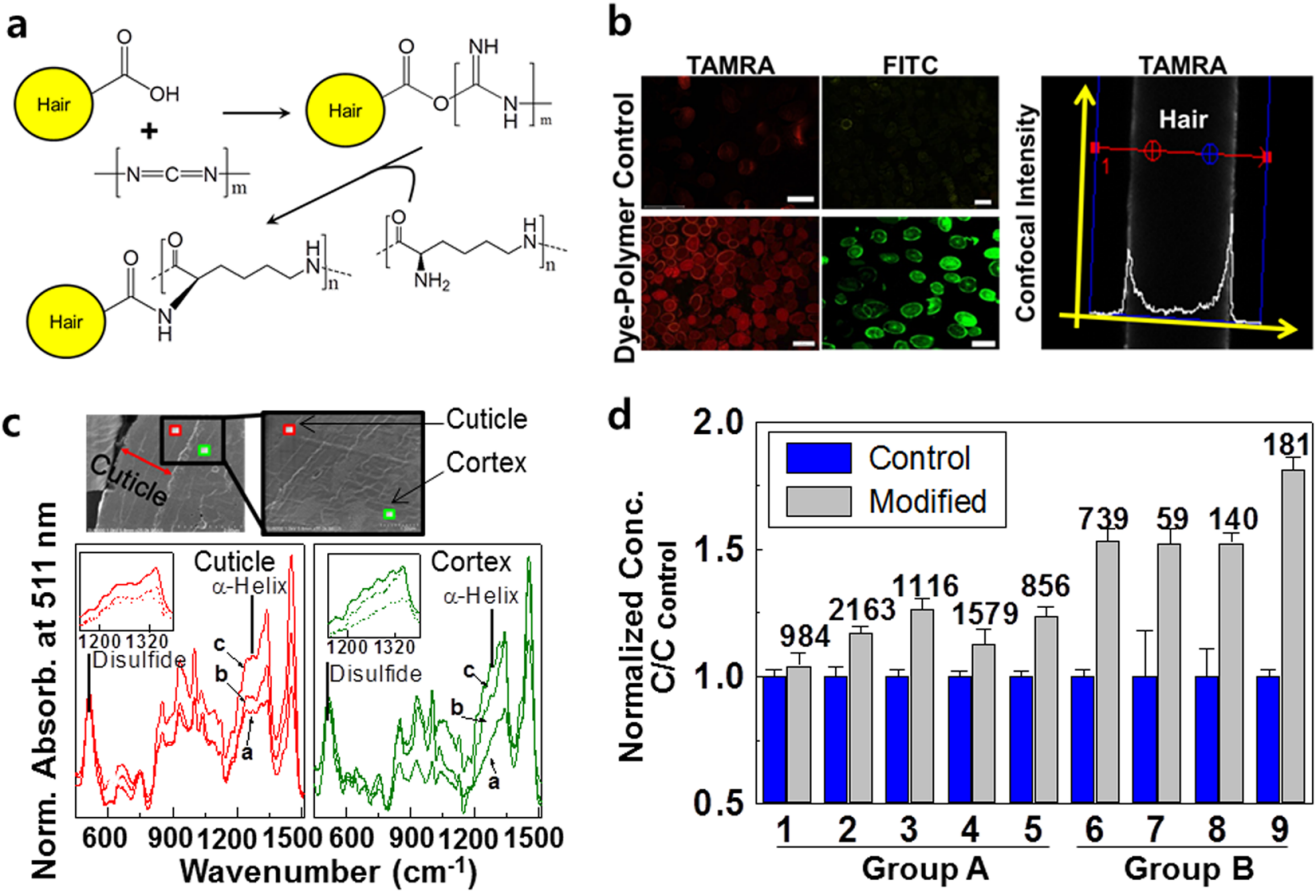
Effect of internal modification of hair on the properties of lipid loss in response to surfactant. (**a**) Schematic diagrams depicting the process of internal modification. (**b**) Fluorescence images (left) of TAMRA-labeled polylysine and FITC-labeled one showing penetration into the hair treated with 5% polylysine and 5% PCI overnight. Scale bars represent 100 μm. The bright color represents polymer penetrated into hair. Confocal microscopic intensity from TAMRA-labeled polylysine (right) is represented by white curve. Data acquisition for the confocal signal by z-stack was performed between blue lines. Hair was treated with TAMRA-labeled polymer for 30 min. (**c**) SEM image of microtomed hair and Raman spectra after immersion in pure water (**a**) and a solution containing 5% polylysine only (**b**), and after sequential immersion in each solution containing 5% polylysine and 5% PCI (**c**). (**d**) The concentration of the remaining lipids after washing the internally modified hair by rubbing 10 times. The normalized concentration was calculated based on the average concentration of unmodified hair (control). The numbers represent the actual concentrations (μg/g hair) of lipids extracted from internally modified hair. The numbers on the x-axis represent lipid described in Table 1.

On the basis of this carbodiimide chemistry, it is assumed that amine compounds form a cross-linkage with the carboxylic acids of the hair, as depicted in Fig. 3a. Polylysine can be used as the primary amine, and it (Mw~1500) was employed for the internal modification in this study. Although carbodiimides have been proven to be nontoxic in cells^21^, we considered that carbodiimides could be insufficiently safe for cosmetic applications. Thus, in order to more clearly remove the toxic potential, we used polymeric carbodiimide (PCI) with an average molecular weight of 400 rather than monomeric carbodiimide.

Several studies have been published on the penetration of fluorescein-labeled polymer into human hair using fluorescence microscopy^22,23^. Experimental proofs of the penetration of PCI and polylysine into the hair are provided in the Fig. 3b. The penetration of TAMRA (5-carboxytetramethylrhodamine)-labeled polylysine into the hair was illustrated in the cross-sections. Since tryptophan in an untreated control hair also can generate fluorescence at lower than 500 nm^24,25^, we detected the emission at 594 nm. Thus, there was no fluorescence signal from hair itself (data not shown). To further visualize the penetration of PCI, FITC (fluorescein isothiocyanate)-labeled the polymers was also detected due to its higher quantum yield^26^. A spectrum from a confocal microscopy corresponding label image is shown in Fig. 3b (right). We used z-stack mode to image deep into normal hair shaft without performing section. At ~10 μm deep into the hair, the fluorescence was still strong. At the images in Fig. 3b, the fluorescence signals of TMARA dye and FITC dye were clearly detected inside hair. From those results, it is obvious that the polymers in mainly localized on the cuticle with some additional penetration into the cortex.

To confirm successful internal modification, Raman spectra were measured (Fig. 3c and Supplementary Fig. 10). The band at approximately 511 cm^−1^ indicates the presence of a disulfide cross-linkage and is present in all analyzed samples. Since all hair samples had not been damaged by oxidizing agents, the amount of the disulfide group would probably be similar. Thus, all spectra were normalized to the band height at 511 cm^−1^. The band at 1273 cm^−1^ in curve (a) owes most of its intensity to amide III region of the hair due to the CN and NH bonds and can be assigned to the α-helix^27^. The bands at 1273 cm^−1^ in the Raman spectra of (b) and (c) were significantly stronger than that in (a). These spectra indicate that the treated hair at the locations of both the cuticle and the cortex has both α-helical protein and polylysine contents, mainly associated with the NH bending vibration and the CN stretching vibration. The spectral intensity at 1273 cm^−1^ in (b) was relatively lower than that in (c), which may imply less durability after washing. Therefore, the overall results demonstrate the successful conjugation of polylysine to hair through carbodiimide chemistry.

The amount of lipid lost from internally modified hair was analyzed by GC/MS (Fig. 3d). The loss of myristic acid was not significantly affected by whether the hair was internally modified. However, the amount of other fatty acids and cholesterol (group A) remained 12.6 to 26.5% higher after washing with surfactant. Interestingly, most of the amounts of squalene and wax esters (group B) were not lost by washing after internal modification. The concentrations of squalene and wax esters remaining after washing were 52.0 to 81.3% higher than those of the control group. In conclusion, the lipids in group A were highly affected by surface modification, whereas the lipids in group B were influenced by internal modification.

## Discussion

The rubbing method, which is one of the washing methods we used, was the same as the actual polishing action conducted during shampooing. We washed the hair by soaking it for 10 min or rubbing it 10 times in almost all experiments. As the washing time or the number of rubbing cycle increases, the amount of loss of the lipids increases. However, the degree of increase was reduced from about 10 min-soaking or 10 times-rubbing (Supplementary Fig. 11). For this reason, we decided to wash the hair by soaking for 10 min or rubbing 10 times.

Considering the pattern in which lipids were removed from the hair, we have classified lipids into two groups: the amphipathic (relatively less hydrophobic) group A and the hydrophobic group B. This classification was based on the solubility of the lipids in water (Supplementary Table 1). Actually, when water was added to the solvent in the extraction step, the amount of lipids in group A to be extracted was increased, whereas lipids in group B was not affected (Supplementary Table 1). During the immersion washing, the presence of surfactants contributed significantly to the loss of fatty acids and cholesterol (group A). In the case of squalene and wax esters (group B), the loss caused by rubbing was much greater than that by immersion. Additionally, the statistically significant difference between the amount of extracted lipids of group A after immersion and rubbing decreased with decreasing solubility in water. This finding means that the amount of lipids lost by immersion is clearly related to the solubility of lipids in water.

The rubbing method implies the application of a shear force because rubbing involves pressure, which can lead to effective surfactant penetration into hair. If so, lipids would easily accumulate in the core of surfactant micelles inside the hair. As shown in Fig. 1b, the amount of group B lipids lost by rubbing was significantly greater than that by immersion. This result is the first reason why we speculated that the loss of lipids in group B was due to the penetration of surfactants.

To study the penetration of surfactants, it is necessary to consider the shape and size of surfactant micelles. A mixture of anionic and zwitterionic surfactants undergoes a transition from a spherical to a cylindrical micelle at 150 mM^28^. Since the surfactant concentration was less than 10 mM in this study, the geometrical shape of micelles would be spherical. The average size of micelles composed of ditetradecyldimethylammonium (C_38_H_80_) when measured directly by cryo-transmission electron microscopy (TEM) was 6 nm^29^, and the typical sizes of micelles are known to be 4.8 nm for sodium dodecyl sulfate and 5.2 nm for cocamidopropyl betaine^30^. The micellar diameter of SLES as measured was 1.4 nm (Supplementary Fig. 12). Indeed, many studies have reported the penetration of surfactants into hair. Surfactants can penetrate the endocuticle and the CMC, and the scale-lifting phenomenon could even occur in the cuticle layer^2^. An energy-dispersive X-ray spectroscopy (EDX) imaging study using cross-sectioned hair showed that micelles of chlorine surfactants existed in the whole area of the hair, but most were present in the outer part^31^. This information suggests that the movement of micelles into the hair is possible.

To confirm that the micelles of surfactants inside the hair cause lipid loss, hair was filled with a cross-linking component using a carbodiimide reaction. Internal filling increases the density of CMC and may restrict the movement of micelles inside hair. The internal modification inhibited the loss of lipids in group B and did not effectively block the loss of group A lipids. In other words, the lipids in group B were more affected by the penetration of micelles than the lipids in group A.

Hair was internally modified when immersed in a solution containing 5% polylysine and 5% PCI, and then washed 10 times. The water absorption of the hair makes hair swell up to 15% in diameter^32^. Because hair was contained in water with a pH value of higher than 10, ionization of diacidic amino acid and hydrolysis of keratin occur and the hair was inevitably swelled^2^. Therefore, the entangled PCI and polylysine can penetrate into the swelled hair. This capability can be also deduced from previous studies demonstrating that keratin-peptide complex, cellulose polymers (Mw~250,000) and polypeptides (Mw~10,000) can penetrate hair^22,33^. Polyethyleneimine and urea (Mw~1200) are known to penetrate to a depth of 6.95 μm, and isocyanate (Mw < 400) penetrated the entire hair. Hence, it is presumed that PCI and polylysine also penetrated into the inside of the hair, resulting in the internal modification of the hair.

Recently, 3-aminopropyltrimethoxysilane (APTES) in a sol-gel system was reported to undergo a reaction between the hydroxyl group of hair and the silanol moiety of APTES^34^. Filling the hair with APTES caused an increase in tensile strength^34^. However, filling the hair with APTES did not inhibit the loss of lipids by surfactants (Supplementary Fig. 13). Its lower molecular weight (Mw ~ 179) and the incomplete binding of silane coupling inside hair may not reduce the volume inside hair sufficiently to affect micelle movement, and this condition may cause the lack of protective effects for hair lipids.

The lipid loss in group A was significantly reduced by the surface modification consisting of deposition of positively charged polymer on the hair surface, whereas the lipid loss in group B was still occurred even after the surface modification. The reason is presumably that most of the lipids in group A may be removed from the surface through a roll-up mechanism rather than from the interior of hair. When the cationic polymer was adsorbed to the surface of hair, the polarity of the surface increased. This induced lower viscous modulus (Fig. 2b) and interfacial tension between the hair and group A lipids^35^. Due to the lower interfacial tension, the wettability of lipids in group A on the surface of hair increased, and the roll-up phenomenon should be reduced. As a result, the surface modification effectively prevents lipid loss through the roll-up mechanism. Obviously, the internal modification retains lipids in group A because the carbodiimide reagent also creates hydrophilic coating on the surface. The fact that the lipids in group A are more affected by the surface modification and are less affected by the internal modification provides a possibility that the components in group A are mainly lost through the roll-up mechanism.

The lipids in group B would also be lost through the roll-up mechanism on the surface of hair. Since these lipids have insoluble in water compared to the group A lipids, the wettability of the lipids to water is low. Therefore, an increase in the charge potential of the surface (i.e., a decrease in the interfacial tension between the lipid and the surface) would not significantly lead to an increase in wettability. Therefore, surface modification would have little effect on the loss of group B lipids. First of all, since the lipids in group B are neutral lipids, surface charge would not be expected to alter their emulsification. Most importantly, as noted above, the loss of group B lipids is significantly blocked by internal modification. Hence, although the lipids of group B can be partially lost due to the roll-up mechanism on the surface, the way they are lost is mainly through surfactant penetration.

The penetration of most surfactants is supposed to occur in the outer part of the hair during washing^31^. However, as shown in Fig. 1c, the loss of the lipids occurred with a uniform distribution. This is undoubtedly because the hair lipids can migrate either through transcellular or intercellular diffusion^36^. The migration of lipids can be occurred due to the equilibrium displacement by the loss of lipids.

**Figure 4:**
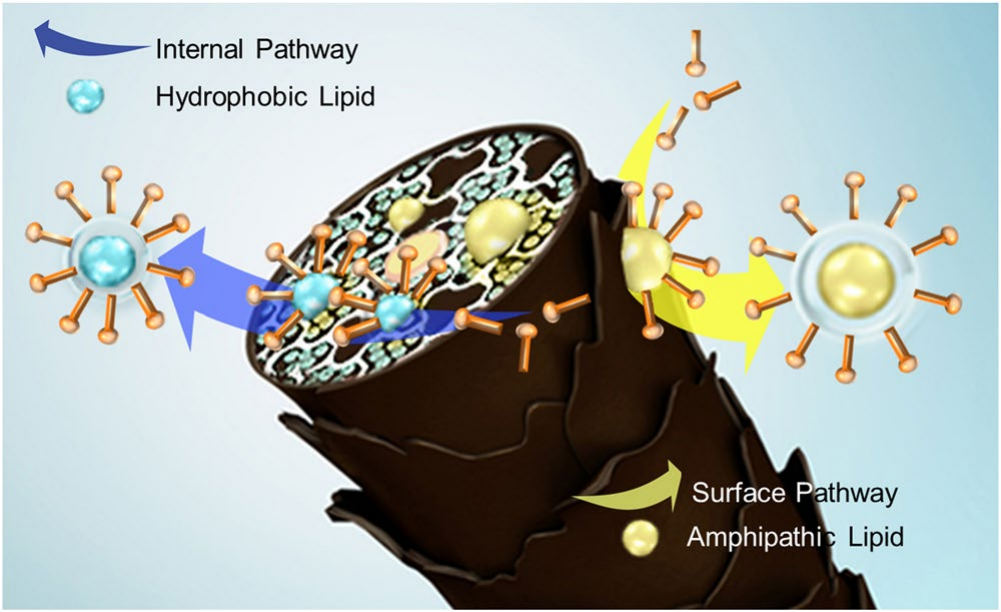
Schematic diagram depicting the pathways of lipid loss by surfactant.

Based on the different patterns between the two groups, we are confident that the mechanism of lipid loss is different for each group. To visually understand the lipid loss caused by surfactant, a schematic diagram is presented in Fig. 4. There are two pathways of lipid loss: the surface and internal pathways. The surface pathway may be controlled by the diffusion of lipids inside the hair and a roll-up mechanism. On the other hand, the internal pathway depends on the penetration of surfactant. Hydrophobic lipids are lost through the internal pathway, and amphipathic lipids follow the surface pathway.

The fact that there are two mechanisms by which lipids are lost means that both must be blocked in order to maintain the health of hair. By simultaneously performing surface and internal modifications, hair damage caused by surfactants can be effectively prevented (Fig. 4). Our study can be utilized to understand and defend the problems of lipid damages by surfactants in various tissues including hair.

## Methods

### Materials

Tetracosane, docosane, palmityl palmitate, stearyl palmitate, myristic acid, palmitic acid, stearic acid, oleic acid, squalene, glyceryl 1,3-dipalmitate (diglyceride), sodium cholesteryl sulfate, o-Terphenyl, and tricosanoic acid were purchased from Sigma-Aldrich (US). Myristyl palmitate, DL-threo-dihydrosphingosine, and DL-erythro-dihydrosphingosine were obtained from Santa Cruz Biotech (US). Cholesterol was obtained from Yakuri Pure Chemicals (Japan). N-(R,S)-Alpha-hydroxyhexadecanoyl-D-erythro-dihydrosphingosine and N-octadecanoyl-D-erythro-dihydrosphingosine were purchased from Matreya (US). 18-Methyleicosanioic acid was purchased from ULTRA Scientific (US).

HPLC grade methanol and hexane were purchased from Duksan (Korea). Chloroform was purchased from Sigma Aldrich (US). APTES was purchased from Sigma-Aldrich (US). Polylysine was synthesized with l-lysine and NaOH in a 1L-reactor, and the molecular weight was approximately 1500. TAMRA and FITC were purchased from Sigma-Aldrich (US).

### Shampoo formulation for surface and internal modification

A cleansing solution used in all tests was formulated by mixing ingredients in water. As a conditioning polymer, 0.5% polyquaternium 10 (PQ-10), a cationic cellulose polymer was used. To increase the delivery efficiency of the conditioning polymer by coacervation, 10% SLES (anionic surfactant) and 3.5% cocamidopropyl betaine (amphiphilic surfactant) were mixed at an elevated temperature of approximately 60 °C. Additional ingredients such as electrolytes and viscosity control agents were added after cooling. The pH value was adjusted to 6.3. Polylysine and polycarbodiimide polymers for internal modification, were also added after cooling. The highly charged cationic cellulose polymers were required for surface modification. They were emulsified at room temperature instead of PQ-10. Newly synthesized polymer had a nitrogen density of 2.0 to 2.8% and a molecular weight of ~800 kD.

### Treatment of hair and surface modification

All experimental protocols with human hair were approved by the LG Institutional Review Board at LG Science Park. All methods were performed in accordance with the relevant guidelines and regulations. Informed consent was obtained in all cases from individual supplier whose hairs were used in this study.

All error bars on graphs in figures were used to indicate the standard deviation and representations of the variability of data from different hair swatches. Hairs longer than 10 cm from the proximal root with one swatch per person were purchased from an individual supplier. These were obtained from twenty females from China or Korea. The hairs had never been treated with any chemical reactions, such as bleaching and permanent waving. The hair swatches were washed with sodium laureth sulfate (SLES) for 1 min and rinsed with water for 2 min. The hair swatch was incubated in a 50% humidity room at 25 °C for 24 h.

Human hair was washed by a cleansing solution with surfactants. Hair was tested by immersion or rubbing in the cleansing solution. At immersion, a shaker was used without shearing of the hair cuticle during washing. The hairs were immersed in glass flasks in a shaking incubator (JeioTech, SI-900R, Korea) rotating at 125 rpm. The temperature of the incubator was 25 °C. 100 mL of cleansing solution was added to 250 mL flask. After shaking, the swatches were rinsed with 37 °C water for 2 min and were blow-dried.

The second section involved rubbing the hair by hand. To wash hair with surfactant, a 1 g hair swatch was prewetted with 1 mL water and then covered uniformly with a solution with surfactants. The foaming was carried out by hand for 15 s. Subsequently, the hair was gently rubbed so that the cationic polymer was absorbed into each hair shaft for 45 s, and the hair swatch was rinsed with water for 2 min. Tap water used in for washing and rinsing flowed from the faucet at a rate of 40 mL s^−1^. After removing excess water, the hair swatch was gently dried with a paper towel. Except for the prewetting step, these steps were repeated several times according to the experimental intent. After washing through the rubbing method, the samples were thoroughly blow-dried.

Surface modification was performed using highly charged cationic polymers. When the hair was treated with a highly viscous solution containing the cationic polymers and blow-dried before washing with SLES, there was no change in the charge potential on the surface of the hair in response to washing. However, when the hair was washed with an SLES solution containing highly charged cationic polymers, the charge potential was effectively increased. To clearly evaluate the effect of surface modification with highly charged cationic polymers, hair was treated with an SLES solution containing a cationic polymer.

### Internal modification

The hair swatch was washed with a 10% SLES solution and transferred to a 200 mL beaker filled with water for cross-linking. Cross-linking was performed in water based on the carbodiimide chemistry. Hair was immersed in the first agent, a 5% solution of polylysine, at 40 °C overnight and then transferred to the second liquid agent, an aqueous solution of 5% polycarbodiimide (PCI), at 40 °C overnight. The pH value of the solution was above 11. Controlled experiments were performed using newly washed hair or the hair that was immersed in an aqueous solution of 5% polylysine at 40 °C overnight. The beakers containing the hairs were cooled to room temperature. Then, the hairs were washed with 10% SLES. The hairs with 5% 3-aminopropyltrimethoxysilane (APTES) were treated in the same manner as performed for polylysine.

### Analysis of lipid content

We have optimized and standardized the method of lipid extraction by altering factors such as the types and ratios of solvents, mass of tissue (hair), extraction period and equipment. The lipid extraction and analysis protocols are described in *SI Materials and Methods*.

### AFM

An atomic force microscope (AFM, XE-100, Park Systems, Korea) was used to evaluate the topography using an NSC36C cantilever (MikroMasch, Germany) in contact mode. A normal spring constant of 0.6 N·m^−1^ was used to determine the compliance of each tip with a typical resonance frequency of 65 kHz. AFM scan was performed for every extraction of lipid after washing process. To examine damaged parts on the cuticle in detail, images were plane-flattened as necessary using the XEI software (v. 4.3.4 build2).

### Determination of ζ-potential and viscous modulus

The ζ-potential of treated hair was determined using the streaming potential (SurPASS3, Anton Paar, Germany). Viscous modulus was determined by a drop shape analyzer (DSA-100, Krüss, Germany). See *SI Materials and Methods* for further details.

### Raman mapping of cross-sectioned hair

Raman imaging was performed with a commercial apparatus (LabRAM HR, HORIBA Jobin-Yvon Inc., Japan). See *SI Materials and Methods* for further details.

### Fluorescence images of hair

The method for tagging the fluorescent dye and the detailed conditions for obtaining the fluorescent image are described in *SI Materials and Methods*.

## Supplementary information

Supplementary Information
