# Supplementary material for: Prevention of lipid loss from hair by surface and internal modification
Source: Sci Rep. 2019 Jul 8;9:9834. doi: 10.1038/s41598-019-46370-x (PMC6614367; doi:10.1038/s41598-019-46370-x)
Supplement: Supplementary file 1 — Supplementary Information [file 41598_2019_46370_MOESM1_ESM.pdf]

Supplementary Information for

Prevention of lipid loss from hair by surface and internal modification

Sang-Hun Song, Jong Hyun Lim, Seong Kil Son, Julia Choi, Nae-Gyu Kang*, Sang-Min Lee*

Sang-Hun Song and Nae-Gyu Kang
Email: sanghunsong@lgcare.com and ngkang@lgcare.com

Materials and Methods

1. Extraction of lipids

The following procedure applies to the extraction of components covalently and noncovalently bound to hair. A 15 cm hair swatch was cut to a length of less than 1 mm with a mass of 50 mg, and the hair residue was transferred to a 15 mL vial filled with 3 mL of extraction solvents. Lipid was extracted merely by isolation with a methylation solvent. The methylation solvent was prepared by the following process. The solvent was mixed so that the ratio of benzene to methanol was 1:3, and 4 g H_2SO_4 was then added to 100 mL of the mixture. After blending for 3 h, 10 g Na_2SO_4 was added, and the blending was continued for another 3 h. The vial was tightly covered with a Teflon cap, and the vial was incubated at 80 °C for 4 d. The standard configuration for the same solvent was placed in the same vial to observe whether the solvent evaporated. The vial was cooled to room temperature, and 2 mL n-hexane was added to the solvent. The fraction was shaken vigorously. After several minutes for complete separation and clarification, the system was separated into two layers. The supernatant containing the extracted lipids was collected. This treatment was repeated three times, and the supernatant of three consecutive collections was evaporated in a cooler to a final volume of 2 mL.

2. Extraction of noncovalently bound lipids

Hair residue was prepared in the same way for covalently and noncovalently bound lipids. A 15 cm hair swatch was cut to a length of less than 1 mm with a mass of 250 mg, and the hair residue was transferred to a 50 mL vial filled with extraction solvents. Each 250 mg sample of hair was homogenized in a 30 mL solvent with a 2:1 chloroform:methanol mixture by sonication for 3 h. The hair residues were suspended for 9 h, and the solvent containing the extracted lipids was decanted to a new vial. Subsequently, a new solvent (30 mL) of a 1:1 chloroform:methanol mixture was added to the hair residue, sonicated for 3 h and suspended for 9 h. After decanting

the solvent to the vial, a new solvent with a 1:2 mixture of chloroform:methanol was added to the hair residue and suspended for 9 h after sonication for 3 h. After the third solvent was collected in a vial, the final solvent containing a 18:9:1 mixture of chloroform:methanol:water was added to the hair residue, sonicated for 3 h and suspended for 18 h. The solvent was decanted to one vial, and the hair lipids were containted in the final 120 mL solvent. The supernatant of four consecutive collections was evaporated in a cooler (JeioTech, hx-03, Korea) to a final volume of 3 mL.

3. Analysis of lipid concentrations

For the gas chromatography/mass spectrometry (GC/MS), samples were analyzed on an Agilent (7890A GC System, US) gas chromatograph coupled to an Agilent detector (5975C MSD System, US). 1 μL of each sample was injected into the gas chromatograph inlet. The injector temperature was maintained at 230 °C during the analysis while the ion source temperature and interface temperature were 250 and 300 °C, respectively. Separation was performed on an HP-5ms UI column (30 m × 0.25 mm × 0.25 μm) using helium as the carrier gas. It was programmed to maintain an initial column temperature of 80 °C for 1 min, then change to 320 °C at a rate of 6 °C min^{-1} and hold for 20 min at this temperature. Injection was performed using a single tapered 4 mm glass inlet liner packed with silanized glass wool in a programmed split mode. Before injection, the carrier gas flow was set to 9 mL min^{-1} with a split value of 5:1. After injection, the split value was set to 5:1, and the carrier gas flow was set to 1 mL min^{-1}.

The mass spectrometer was operated in an electron impact mode at an ionization voltage of 70 eV. Mass spectra in the full scan mode were recorded in a mass range of 50-500 amu. Selected ion monitoring (SIM) was carried out by monitoring m/z 228 for myristic acid, m/z 256 for palmitic acid, m/z 69 for oleic acid, m/z 284 for stearic acid, m/z 85 for docosane, m/z 85 for tetracosane, m/z 74 for 18-MEA, m/z 121 for squalene, m/z 386 for cholesterol, and m/z 257 for myristyl palmitate, palmityl palmitate, and stearyl palmitate. Peak identification was based on

comparison with standards for retention time and mass spectral fragmentation. m/z 230 for o-Terphenyl as an internal standard was added to the lipid solution. 100 mg of each reference substance was dissolved in 10 mL of a 2:1 mixture of chloroform and methanol. The concentration of all mixtures of reference substances was 1000 mg L^{-1}. 10μL o-Terphenyl (2000 mg L^{-1}) was added to 1 mL of the extracted lipids.

GC-flame ionization detector (FID) analysis with an Agilent detector (GC3461A system) was performed in the same way as performed for GC-MS. All high-performance liquid chromatography (HPLC) experiments were performed with an Agilent 1260 Infinity II instrument (Agilent Technologies, Waldbronn, Germany) equipped with a UV-VIS diode array detector (DAD), an Agilent Technologies 6410A Triple Quadruppole mass spectrometer, and a multimode ion source (MMI). Data analysis was carried out using Agilent MassHunter Workstation Software B.01.03. To 200 μL extracted solution containing lipids, 1 mL of 1 % 2,4-dibromoacetophenone (v/v) was added. Then, 1 mL of 1% acetonitrile was added. The reaction mixture was capped, mixed with a vortex mixer and stored at 80°C for 1 h. After cooling, the final solution was filtered and stored at room temperature until HPLC analysis.

Matrix-assisted laser desorption ionization–tandem time of flight mass spectrometry (MALDI-TOF/TOF MS) analysis was performed in the positive ion reflectron mode using an ultrafleXtreme system (Bruker Daltonics). The samples were analyzed as described previously [1].

4. Raman mapping of cross-sectioned hair

Because melanin in black hairs can affect Raman signals, European white hair (Jehyun trading, Korea) was used for Raman analysis. White hair was placed at -120 °C and cut with an ultramicrotome system (Leica EM FC7) at a cutting speed of 0.2 mm s^{-1}. Hair was cut on one side because a variation in signal can be caused by variations of sample thickness. The use of tape (3M Scotch Cat. 810D) for embedding improved the quality of cross-sections. Images were

recorded with a MZ6 stereo microscope (Leica). Scanning electron microscope (SEM) images of hair were obtained using a Hitachi S-4800 instrument (Hitachi, Japan).

Raman imaging combining spectral and spatial information at the same time was performed with a commercial apparatus (LabRAM HR, HORIBA Jobin-Yvon Inc., Japan). A semiconductor diode near-infrared laser operating at λ = 1064 nm was used as the excitation source and delivered a laser power of 2.5 mW measured at the cross-sectioned hair. The Raman images were obtained using a Raman point mapping scan. Light from the illuminated spot was collected by an optical lens and sent through a monochromator with an optical filter to block Raleigh scattering. The Raman signal was focused through a 500 μm wide slit and dispersed by a diffraction grating of 1800 grooves per millimeter onto a deep depletion InGaAs detector (Horiba) of a size of 30 × 30 pixels with 1 μm × 1 μm area. The exposure time per pixel was 40 s, and the scan was repeated 10 times. The distribution of Raman spectra was analyzed using a software program (Labspec 6.5.2.5).

5. Determination of the ζ-potential

The ζ-potential of treated hair was determined using the streaming potential (SurPASS3, Anton Paar, Germany). A 0.5 g bundle of treated hair was placed in a cylindrical cell without cutting. Measurements were conducted in 1 mmol L^{-1} KCl as a supporting electrolyte. All measurements are performed with three samples for the hair bundle.

To determine the ζ-potential of the hair surface, the streaming current coefficient $\frac{dU_{str}}{d\Delta p}$ was measured at 299 K and pH 5.8. The ζ-potential was calculated from the electrophoretic mobility measured using the Helmholtz-Smoluchowski (equ. 1).

$$\zeta = \frac{dU_{str}}{d\Delta p} \times \frac{\eta}{\varepsilon \times \varepsilon_0} \times \kappa \quad \text{(equ. 1)}$$

where κ, the electrical conductivity, is 0.025 S m^{-1}, and η and $\varepsilon \times \varepsilon_0$ are the viscosity and dielectric permittivity of the electrolyte solution are 8.79×10^{-4} Pa·s and 78, respectively.

Since the Ag/AgCl electrode is affected by the cationic polymer, the non-zero voltage offset at zero pressure was due to the polymers in the hair sample (Fig. 2a). The streaming current coefficient of positively charged hair was higher than that observed in the control (Fig. 2A). The measured streaming current coefficient of hair changed almost linearly at pressures ranging from 2.0 to 4.5 mbar. PQ10B, PQ10C and PQ10D were found to provide appropriate cationic properties. The averaged ζ-potential was determined to be -16.3 mV for the control using (equ. 1). A hair has anionic surface due to its low isoelectric point,[2] and the hair treated with a cleansing solution containing general cationic polymer also had negatively charged surface even in repetitive treatments (data not shown). Surprisingly, the averaged ζ-potential was determined to be 17.1 mV, 25.1 mV and 31.6 mV for the PQ10B, PQ10C and PQ10D, respectively.

6. Viscous modulus

To understand viscous modulus and ζ-potential pattern after coating the hair surface with PQs, surface tension, γ was measured by drop shape analyzer, DSA-100 (Krüss, Germany) using pendant drop method.[4] The surface viscosity, μ was obtained from equ. 2.

$\mu = \frac{d\gamma}{dlnA} \cdot \frac{1}{f}$ (equ. 2)

where A is interfacial volume dilatation, and f, the frequency for deformation of droplet of 0.5% PQs, was 0.1 Hz. All experiments were averaged with repetitive (at least three) cycles.

7. Dye tagging and fluorescence images of hair

To perform the fluorescence quenching experiments, carboxytetramethylrhodamine (TAMRA) dye was added to a 5% aqueous solution of polylysine. Then, the TAMRA-labeled polylysine was purified by filtration and dialysis for 2 d. The purified TAMRA-labeled polylysine solution to be lyophilized was frozen in liquid nitrogen and applied to a Bondiro (IlshinBio, Koorea) model 8

freeze-drier at a pressure of approximately 50 mTorr and a condenser temperature of -80 °C. The lyophilization was performed for 1 week.

Small hair swatches consisting of 20 fibers of 5 cm length were socked in an aqueous solution containing 5% TAMRA-labeled polylysine or an aqueous solution containing 5% TAMRA-labeled polylysine and 5% PCI for 24 h each. Cross-section of the hairs for fluorescence measurements was prepared in the same way for samples for Raman measurements.

Fluorescence image for TAMRA was acquired using a fluorescence microscope (Leica DM1RB, Leica Microsystems, US). The fluorescence signals of hair fibers were observed at 580 nm after excitation at 515 nm (N2.1 filter). Dye tagging with fluorescein isothiocyanate (FITC) was performed in the same way as was performed for TAMRA. In the case of FITC, fluorescence signal was collected at 525 nm with excitation at 470 nm (EVOS2 filter) in another fluorescence microscope (Evos Light cube, Thermo Fisher, US).

The fluorescence signal inside hair was imaged with a confocal microscope (LSM 710 Confocal Microscopy, Carl Zeiss, Germany). The fluorescence was excited with the 543-nm line of Zeiss LSM 5 Exciter, detected using a 560-nm long-pass emission filter, and quantized with a resolution of 100 nm depth.

8. Micellar size of SLES

Critical micelle concentration (cmc) of SLES was determined by a tensiometer (K100C, KRUSS, Germany), and the cmc as measured was 0.428 mM. The size of micelle in the SLES solution (c=3 mM) was monitored by dynamic light scattering method using a zetasizer (ZEN3600, Malvern Instruments, UK).

Supplementary Table 1. The amounts of lipids from the extraction of hair (μg/g hair). The ± sign indicates the standard deviation. The analyses from (a) to (e) were performed by GC/MS. The hair lipid in (a) was extracted successively with 30 mL portions of 2:1, 1:1, and 1:2 ratios of $CHCl_3$ to CH_3OH, and the hair residue was homogenized with a 18:9:1 mixture of $CHCl_3$:CH_3OH:H_2O. Those steps were performed with sonication for 3 hours each, and suspension was performed between different solvent mixtures for 9 hours. In (b), the extraction by the fourth solvent that has water was not performed. (c) was performed by shaking instead of sonication. All samples were extracted with 250 mg of hair, but (d) was performed with 900 mg of hair. (e) was performed for 4 days. In the case of (e), sonication was performed for 3 hours each, and the sample was suspended for 21 hours each. The lipid extractions of (f) and (g) were performed in the same way as was performed for (a), but the sample was analyzed by GC/FID and LC/UV, respectively. The analytical results are the average from 6 samples.

**Group**	**Species**	**(a)**	**(b)**	**(c)**	**(d)**	**(e)**	**(f)**	**(g)**	**Solubility in water (mg/L)**[5]
		-	water	method	amount	period	GC/FID	LC/UV	
**A**	**Myristic acid**	1285 ±34	1191 ±49	1125 ±78	1346 ±65	1315 ±77	1202 ±16	1028 ±49	22 (at 30℃)
	**Palmitic acid**	2763 ±55	2733 ±75	2560 ±172	2883 ±58	2836 ±54	2522 ±29	2917 ±74	0.04 (at 25℃)
	**Stearic acid**	1274 ±23	820 ±37	1104 ±152	1116 ±51	1224 ±57	1137 ±21	1326 ±36	0.597 (at 25℃)
	**Oleic acid**	2096 ±38	1754 ±69	1458 ±77	2004 ±95	2285 ±51	1946 ±20	1862 ±46	0.01 (at 25℃)
	**Cholesterol**	891 ±26	811 ±58	852 ±96	899 ±14	894 ±16	768 ±23	846 ±63	0.095 (at 30℃)
**B**	**Squalene**	874 ±17	881 ±52	771 ±47	958 ±21	875 ±14	962 ±5	902 ±26	Insoluble
	**Myristyl palmitate**	60 ±3	57 ±11	52 ±15	61 ±13	65 ±16	34 ±3	-	Insoluble
	**Palmityl palmitate**	144 ±5	133 ±39	98 ±23	149 ±13	160 ±7	109 ±7	-	Insoluble
	**Stearyl palmitate**	243 ±12	239 ±24	125 ±26	248 ±16	249 ±27	195 ±7	-	Insoluble

This section aims to determine the method for extracting the maximum amount of lipid. Here, we present a comparison to define the best method whereby a high concentration of lipids can be extracted in a 2 day process (Supplementary Table 1 (a)). The amounts of lipids obtained by various extraction methods are shown in Supplementary Table 1. Additionally, the lipids are divided into 2 groups based on the assumed hydrophobicity.

To determine the composition of solvents, hair mass, period of extraction, and analytical method, the amounts of lipid were determined in repeated analyses. The mixture of chloroform and methanol is conventionally used as the solvent for lipid extraction in histological studies of hair.[6-9] Different mixtures with varying ratios of chloroform to methanol, which are used to effectively extract lipids that have different polarities, are required to achieve the maximum yield for the extraction. Therefore, 250 mg of hair residue was sonicated in the chloroform:methanol series of 2:1, 1:1, and 1:2 for 3 hours each with suspension for 9 hours. The recent method of Wu et al.[7] maximized the extraction quantity via $CHCl_3/CH_3OH = 2:1$, but the study extracted only the squalene fraction from the total lipids. To increase the extraction of polar lipids, water was added in the last procedures. When water was excluded from the solvent for extraction in Supplementary Table 1 (b), we obtained dominantly less group A lipids in repeated experiments. However, the group B lipids mostly did not show a difference even when water was not added to the solvent. Finally, the results indicate that three extractions and one subsequent extraction with water were sufficient for solvent extraction of hair lipids.

Extraction was performed by shaking, as indicated in Supplementary Table 1 (c). Fewer lipids were extractable, and the standard deviation (SD) was larger for the group A lipids. Considering the empirical results, we decided to use sonication during extraction.

Additionally, the lipid was extracted from 50 mg and 900 mg of hair. In the case of 50 mg, wax esters had a significantly higher SD due to its lower concentration. The amounts of extracted lipid increased for 900 mg of hair, as indicated in Supplementary Table 1 (d). However, the amounts of lipid obtained from 250 mg and 900 mg of hair mostly show no significant difference. This finding shows that an excess of hair does not guarantee a higher concentration (per gram of hair) of extracted lipids.

Very similar results were found for 2-day extraction and 4-day extraction, as indicated in Supplementary Table 1 (e). The period of extraction slightly increased the extraction efficiency of lipids such as myristic acid and palmitic acid, but the amount of stearic acid and squalene slightly decreased in the 4-day extraction. Finally, in this study, we chose the short extraction time (2 days) to minimize oxidative decomposition and to perform experiments economically. Many studies performed lipid extraction for different periods. The method of Duvel [8] required too many exchanges for dissolving solvent (total of 11 times). The method of Takahashi [9] was rapid but used methylation, which cannot distinguish the noncovalently bound part from the total lipids. The method of Masukawa[6] was performed for 4 days. Here, a 2-day process has been established with the optimal conditions for lipid extraction.

Next, we selected the analytical tool to determine the lipid concentration. GC/FID had a good ability to quantify the amount of lipids and provide successful analysis with low interference from other substances.[10-11] For this study, GC/FID was inadequate because it was not sensitive enough to detect small amounts of lipids like stearic acid (Supplementary Fig. 3). Additionally, GC/FID did not distinguish tetracosane from 18-MEA due to the peak overlap (Supplementary Fig. 3). Many studies used liquid chromatography (LC) to analyze hair lipids[6, 8, 10, 12-13]; however, HPLC has a limitation when used to analyze cholesterol and 18-MEA.[14] LC/UV cannot analyze hydrocarbons and wax esters, and it is difficult to prepare samples due to derivatization. Finally, GC/MS was employed for analyzing the concentration of lipids. At the end, the concentrations of squalene and cholesterol determined in this study were larger than the 700 μg/g hair and the 410 μg/g hair values reported in other studies.[6, 12]

Supplementary Table 2. Retention times (RT) for the hair lipids. **a** Retention times for the experiments in the Supplementary Figs 1-3, and 6-7. **b** Retention times for the analyses of lipid loss in Figs. 2-3, Supplementary Figs. 5, 11 and 13.

a

	**Compound**	**RT (min)**	**Calibration range (ppm)**
1	Myristic acid	17.5	10-250 (7 points)
2	Palmitic acid	20.8	25-250 (6 points)
3	Oleic acid	23.5	25-250 (6 points)
4	Stearic acid	23.8	25-250 (6 points)
5	Docosane	24.4	1-25 (4 points)
6	Tetracosane	27.1	1-25 (4 points)
7	18-MEA (methyl ester)	27.2	1-25 (4 points)
8	Squalene	32.5	10-100 (5 points)
9	Cholesterol	35.7	25-250 (6 points)
10	Myristyl palmitate	36.1	1-62.25 (6 points)
11	Tricosanoic acid	37.0	1-62.25 (6 points)
12	Palmityl palmitate	38.1	1-62.25 (6 points)
13	Stearyl palmitate	40.0	1-62.25 (6 points)

b

	**Compound**	**RT (min)**	**Calibration range (ng)**
1	Myristic acid	19.29	10-250 (7 points)
2	o-Terphenyl	21.52	25 ng
3	Palmitic acid	22.61	25-250 (6 points)
4	Oleic acid	24.66	25-250 (6 points)
5	Stearic acid	25.44	25-250 (6 points)
6	Squalene	34.10	10-100 (5 points)
7	Cholesterol	37.56	25-250 (6 points)
8	Myristyl palmitate	37.76	1-62.25 (6 points)
9	Palmityl palmitate	39.72	1-62.25 (6 points)
10	Stearyl palmitate	41.60	1-62.25 (6 points)

Supplementary Table 3. Lipid concentrations for different periods of methylation (μg/g hair). Extraction of covalently bound lipids and noncovalently bound lipids. The methylation period of 4 days was selected because the maximum amount of lipids was obtained at 4 days.

Methylation time (h)	Myristic acid	Palmitic acid	Oleic acid	Stearic acid	18-MEA
**3**	1193	3185	2550	647	117
**6**	1205	3179	2490	653	163
**16**	1210	3185	2497	655	192
**48**	1465	5160	5075	1605	236
**120**	1567	4896	4839	1601	240

Supplementary Table 4. Amounts of lipids in hair after washing with SLES and hexane (μg/g hair). The analytical results are the average from 10 samples. The ± sign indicates the standard deviation.

	Sample	Myristic acid	Palmitic acid	Stearic acid	Oleic acid	Squalene	Cholesterol
(a)	Virgin hair	1202 ±26	2522 ±58	1270 ±3	2091 ±17	943 ±109	980 ±17
(b)	SLES for 1 min	1225 ±53	2589 ±85	1267 ±42	2142 ±33	874 ±24	875 ±24
(c)	Hexane for 5 min	1189 ±74	2474 ±104	1237 ±97	2047 ±75	857 ±57	867 ±63
(d)	SLES for 1 min→ Hexane for 5 min[a]	1212 ±46	2485 ±47	1230 ±82	2008 ±53	868 ±81	885 ±53
(e)	SLES for 1 min→ Hexane for 5 min[b]	ND	ND	ND	ND	59 ±1	72 ±3
(f)	SELS for 1 min→ Hexane for 10 min	1180 ±67	2414 ±84	1173 ±85	1790 ±75	849 ±68	879 ±76
(g)	SELS for 1 min→ Hexane for 2 h	1115 ±55	2268 ±67	1077 ±103	1795 ±72	778 ±68	826 ±91

An XPS study confirmed that even after shampooing one time, an appreciable amount of noncovalently bound lipid was removed to a depth of 3 nm.[15] One may think that the lipid loss determined by GC/MS is attributable to the peripheral lipid on the hair surface. To remove the lipids on the surface, hair was washed with SLES and hexane. To remove lipids contaminating the surface, both SLES and the n-hexane were used for washing. [7, 12, 16]

The data for comparable washing of virgin hair with SLES and hexane are shown in Supplementary Table 4. To remove peripheral lipids on the surface of hair, 1 g of as received hair was homogenized with 100 mL of 10% SLES for 1 min and washed for 2 min. Then, the hair was incubated in 100 mL of hexane and successively washed for 2 min (Supplementary Table 4(d)). The samples in Supplementary Table 4(b) and (c) were washed with only SLES and hexane, respectively. GC/MS analysis was conducted on an extracted sample from each of the washing subjects. All data in Supplementary Table 4 are the concentrations determined from the hair, but the hexane 5 min[b] data point (e) is the concentration from the solvent extraction that used hexane for 5 min[a] (d).

The amount of squalene was 943 μg/g hair in as received virgin hair (Supplementary Table 4(a)) and 874 μg/g hair in hair washed with SLES. However, the amounts of C14 and C16 did not decrease from Supplementary Table 4(a) to (b). Washing with SLES one time (b) conserves more lipids than washing with hexane for 5 min (c). When the hair was incubated in hexane for 5 min, the concentrations of lipids were slightly decreased. In spite of the variations in the conditions, there was virtually no change in the concentrations of hair constituents between (c) and (d). These findings indicate that we do not need to use the two together. The amount of lipid decreased with hexane incubation time ((e), (f), and (g)). This finding indicates that the internal lipids might be lost by diffusion from the inside to the outside with longer exposure to hexane. Considering these results, we concluded that the hexane incubation is not needed to protect the internal lipids of hair. Incubation of hexane may be extracting a tiny amount of lipids from the inside of hair. Finally, all hairs were incubated with a 10% SLES solution for 1 min. On the assumption that prewashing with SLES does not extract an appreciable amount of internal lipids, prewashing was carried out.

Supplementary Table 5. Concentration of hair lipids subjected to rubbing wash with SLES and the shampoo formulated with SLES and a cationic polymer (μg/g hair). The analytical results are the average of 6 samples. The ± sign indicates the standard deviation.

**(μg/g hair)**	**(a)** SLES 5 times	**(b)** Shampoo 5 times	**(c)** SLES 10 times	**(d)** Shampoo 10 times
**Myristic acid**	1092±32	1066±34	963±27	938±23
**Palmitic acid**	2384±66	2409±73	1961±59	1934±35
**Stearic acid**	1100±43	1093±35	891±37	879±17
**Oleic acid**	1889±52	1864±43	1592±52	1550±23
**Cholesterol**	830±43	832±42	678±31	660±9
**Squalene**	654±32	649±27	503±3	512±6
**Myristyl palmitate**	45±3	47±3	37±3	35±3
**Palmityl palmitate**	91±3	93±3	72±3	75±3
**Stearyl palmitate**	163±3	165±3	137±3	135±4

Supplementary Fig. 1. Chromatogram of the samples in Supplementary Table 1. (a) Sample A, (b) Sample B, (c) Sample C, (d) Sample D, (e) Sample E, (f) references 50 ppm 18-MEA. Graph: 1. Myristic acid, 2. Palmitic acid, 3. Stearic acid, 4. Oleic acid, 5. Docosane, 6. Tetracosane, 7. 18-MEA, 8. Squalene, 9. Cholesterol, 10. Myristyl palmitate, 11. Palmityl palmitate, 12. Stearyl palmitate.

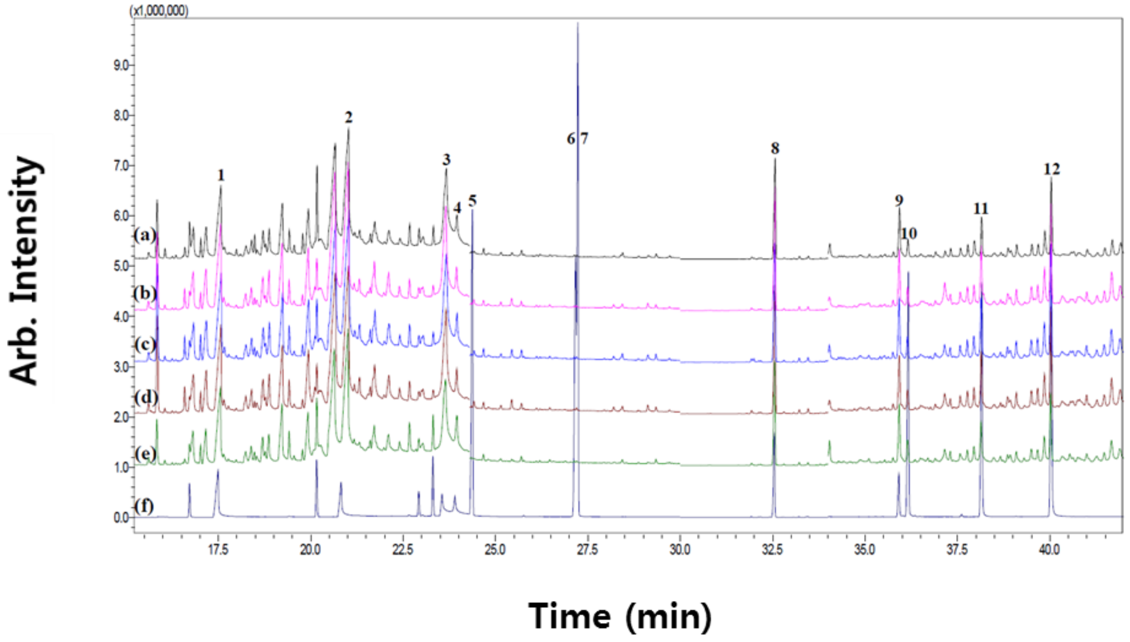

Supplementary Fig. 2. Reference curves. A stock solution of each lipid was dissolved in 2:1 chloroform:methanol at a concentration of 1000 mg/L. The concentrations in the first mixture were 10, 20, 50, 60, 100, 130, and 250 ng for each fatty acid, squalene, and wax ester; the concentrations in the second mixture were 1, 5, 10, and 25 ng for docosane, tetracosane, and 18-MEA.

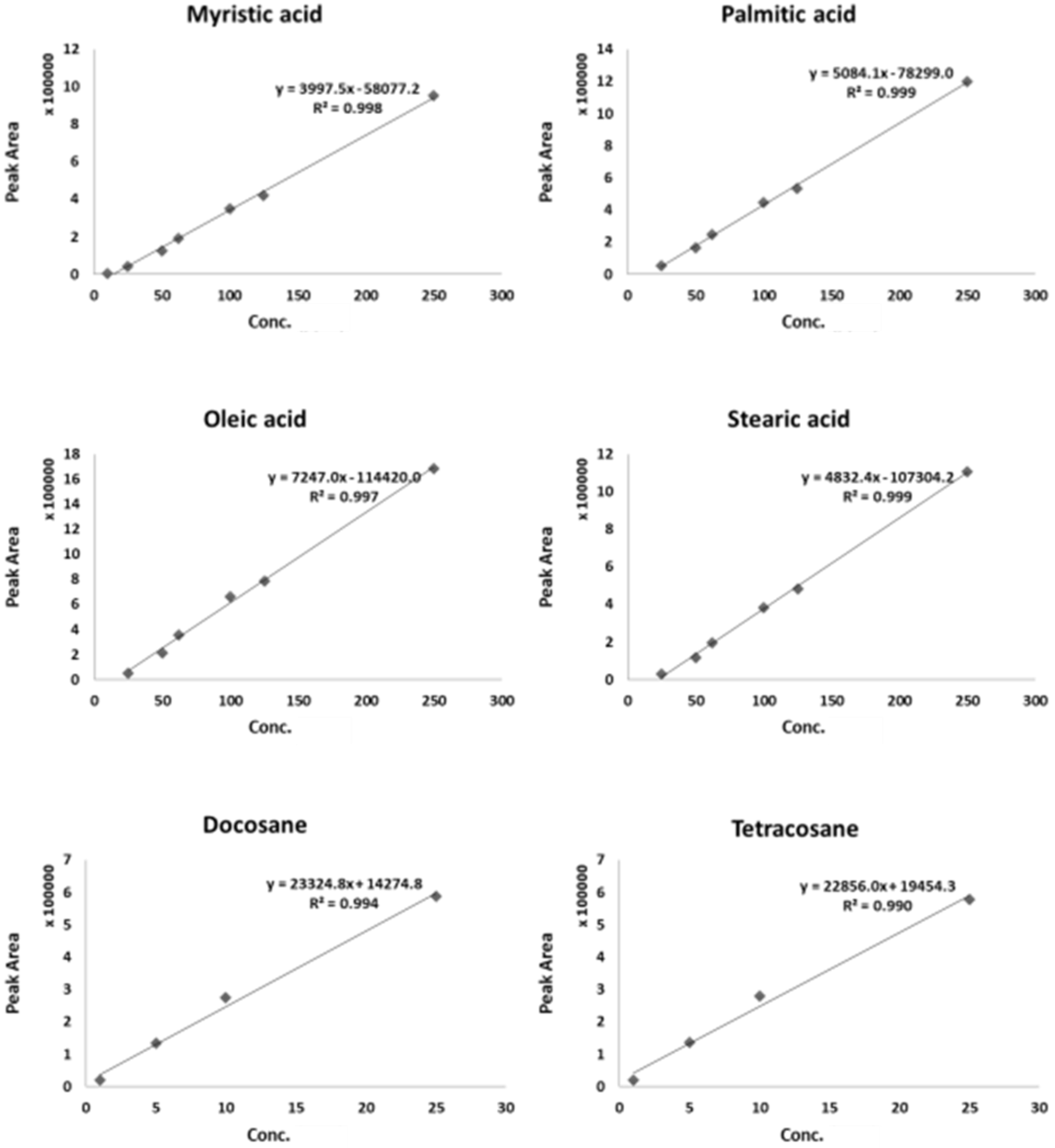

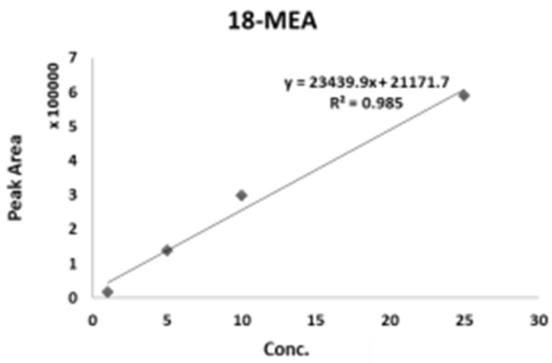
18-MEA
y = 23439.9x + 21171.7
R² = 0.985
Peak Area
x 100000
Conc.
0 1 2 3 4 5 6 7
0 5 10 15 20 25 30

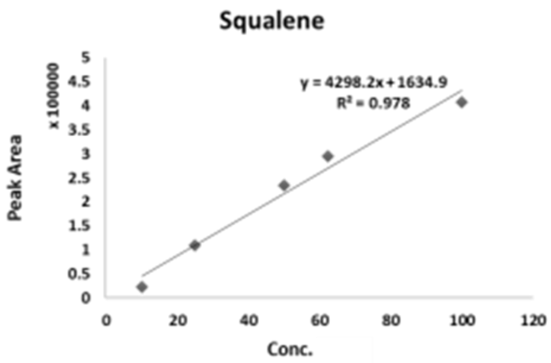
Squalene
y = 4298.2x + 1634.9
R² = 0.978
Peak Area
x 100000
Conc.
0 0.5 1 1.5 2 2.5 3 3.5 4 4.5 5
0 20 40 60 80 100 120

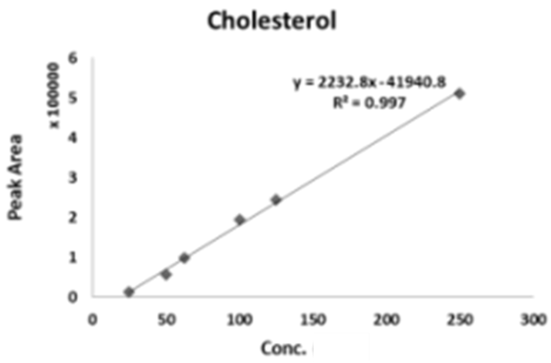
Cholesterol
y = 2232.8x - 41940.8
R² = 0.997
Peak Area
x 100000
Conc.
0 1 2 3 4 5 6
0 50 100 150 200 250 300

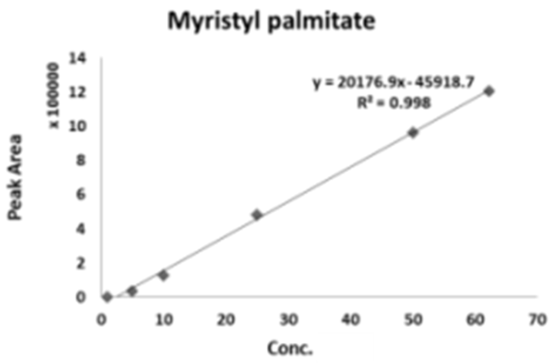
Myristyl palmitate
y = 20176.9x - 45918.7
R² = 0.998
Peak Area
x 100000
Conc.
0 2 4 6 8 10 12 14
0 10 20 30 40 50 60 70

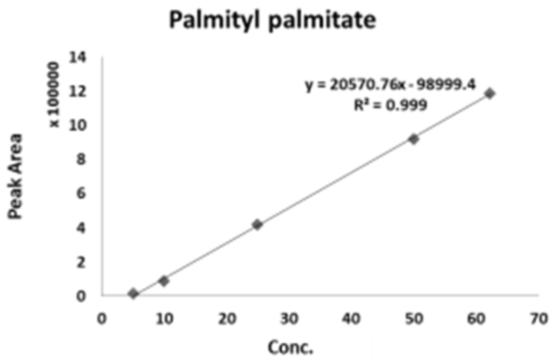
Palmityl palmitate
y = 20570.76x - 98999.4
R² = 0.999
Peak Area
x 100000
Conc.
0 2 4 6 8 10 12 14
0 10 20 30 40 50 60 70

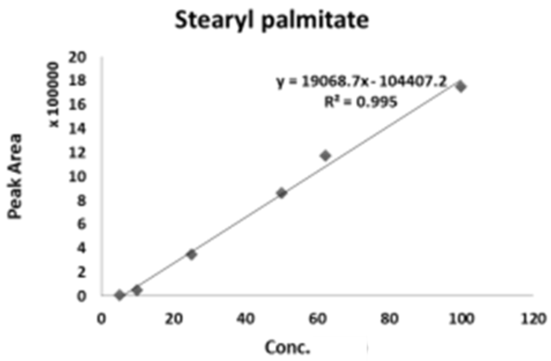
Stearyl palmitate
y = 19068.7x - 104407.2
R² = 0.995
Peak Area
x 100000
Conc.
0 2 4 6 8 10 12 14 16 18 20
0 20 40 60 80 100 120

Supplementary Fig. 3. Comparison of the chromatograms from GC/FID (A) and GC/MS (B). GC/MS is able to distinguish the tetracosane peak from the 18-MEA peak, whereas GC/FID cannot distinguish these peaks. GC/MS is more sensitive for detecting stearic acid than GC/FID. Peak: 1. Myristic acid, 2. Palmitic acid, 3. Stearic acid, 4. Docosane, 5. Tetracosane, 6. 18-MEA, 7. Squalene, 8. Cholesterol, 9. Myristyl palmitate, 10. Palmityl palmitate, 11. Stearyl palmitate

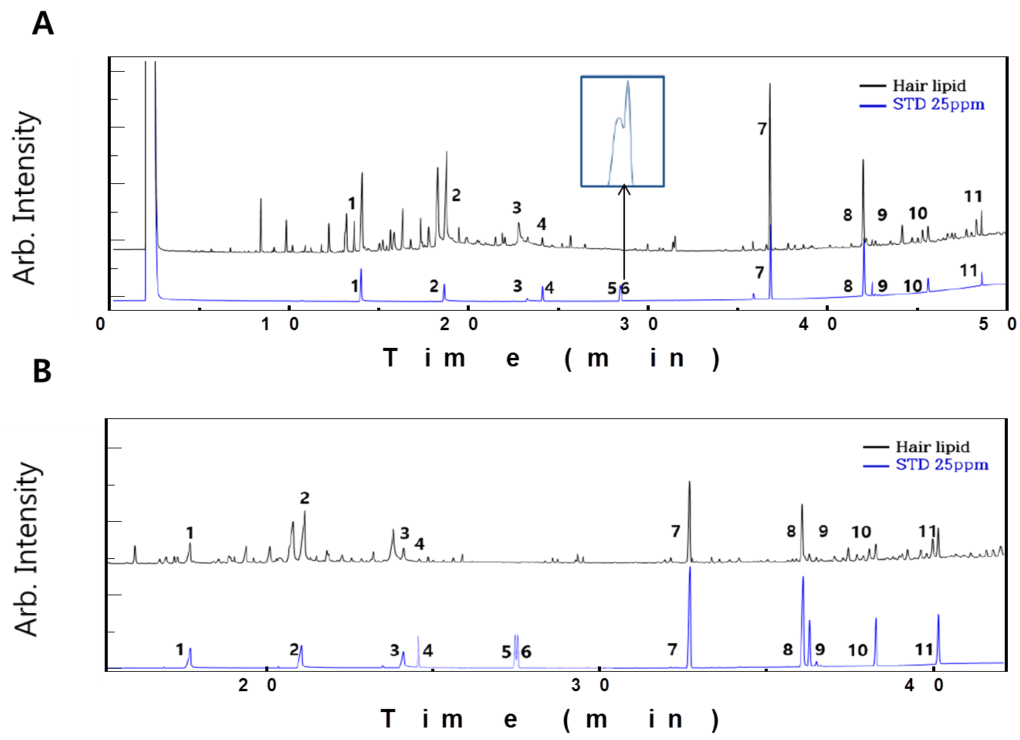

Previously, TLC (thin-layer liquid chromatography) was used to analyze ceramides, an important component in hair.[8, 16] However, in our study, the presence of ceramides was not demonstrated using MS chromatograms and even MALDI-TOF (Matrix-assisted laser desorption/ionization-time of flight mass spectrometry) because either ceramides could not be extracted under our experimental conditions or our sample has a very low concentration of ceramides. Docosane, tetracosane, and eicosane were examined as a family of hydrocarbons and were determined to have low concentrations below 3 ppm. Triglycerides are reported to correspond to only 2% of hair lipids.[2] In addition, the ratios of triglycerides to fatty acids vary by person because personal products chemically alter the contents of triglycerides produced in sebum.[17] Accordingly, ceramides, hydrocarbons, and triglycerides were not studied here.

Supplementary Fig. 4. TOF-MALDI data for ceramides. Five ceramides were used as model compounds for ceramides. (A) Two kinds of sphingosine. Those sphingosines are ceramides, but hydrocarbons were not used: DL-threo-dihydrosphingosine, and DL-erythro-dihydrosphingosine. (B) Three ceramides: N-hexadecanoyl-D-erythro-dihydrosphingosine, N-octadecanoyl-D-erythro-dihydrosphingosine, and N-(R,S)-alpha-hydroxyhexadecanoyl-D-erythro-dihydrosphingosine. For glycerides, it was difficult to detect each component due to the low concentrations. This finding indicates that either the ceramides exist in hair at concentrations below 10 μM (limit of detection) or ceramides do not exist in our hair sample.

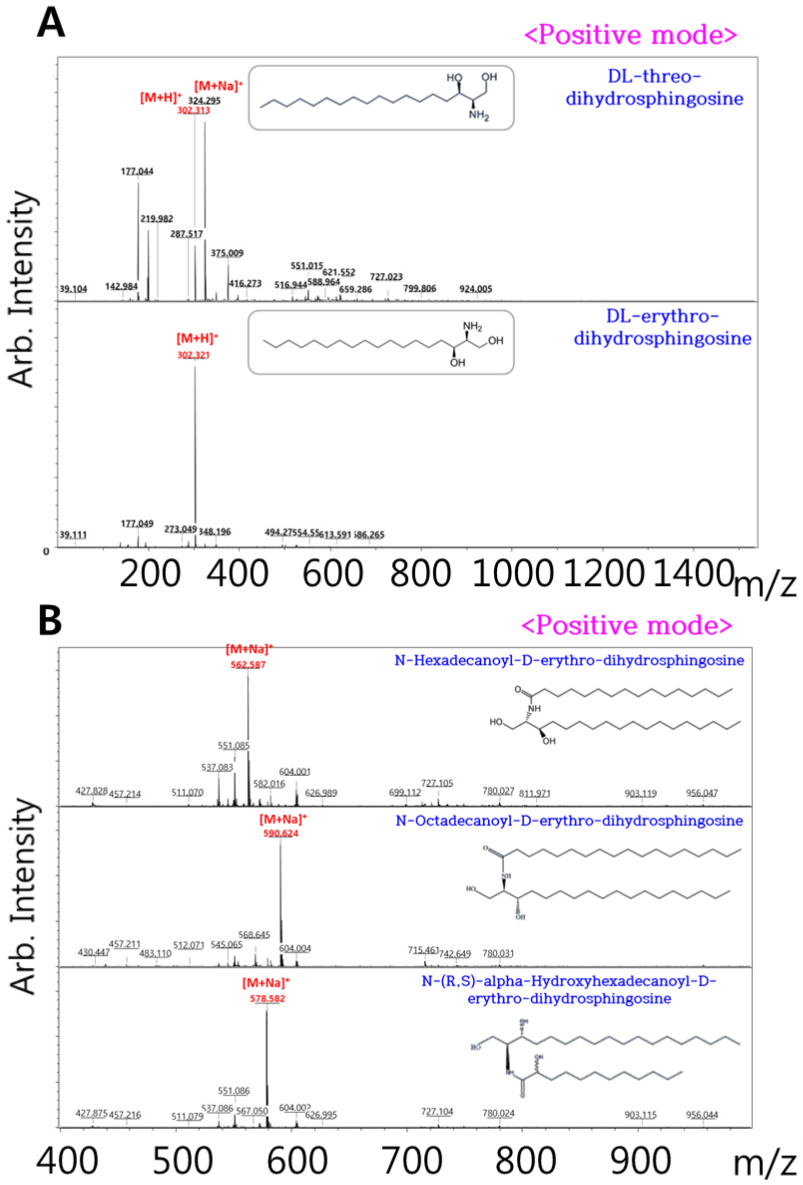

Supplementary Fig. 5. Retention time (RT) for the hair lipids with the internal standard o-Terphenyl. (A) Standard materials. (B) Hair sample. Peak: 1. Myristic acid; 2. o-Terphenyl; 3. Palmitic acid; 4. Stearic acid; 5. Oleic acid; 6. Cholesterol. 7. Squalene; 8. Myristyl palmitate; 9. Palmityl palmitate; 10. Stearyl palmitate.

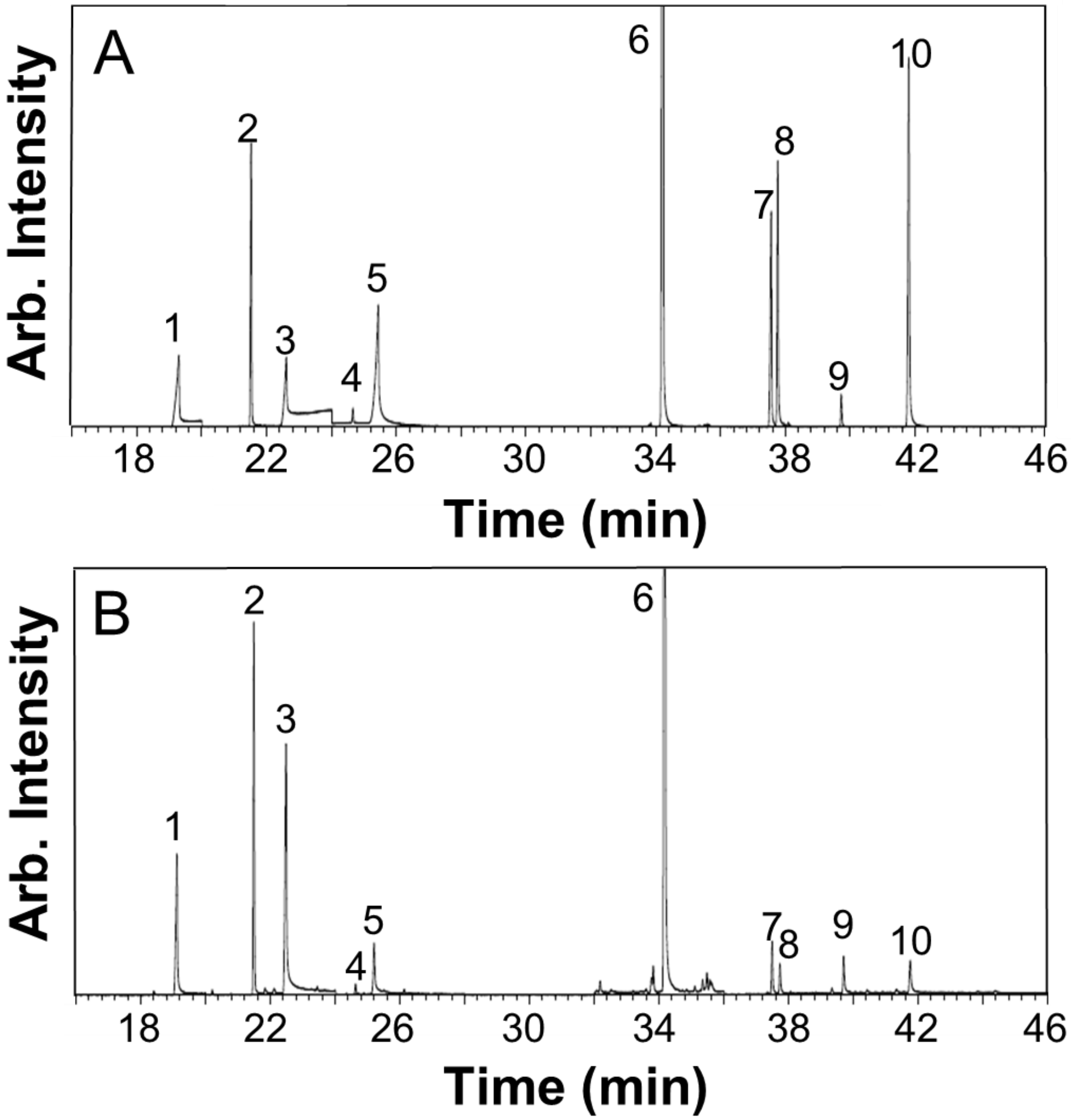

Supplementary Fig. 6. GC/MS SIM chromatogram of standard materials after methylation for Supplementary Fig. 7. These results involve not only fatty acids but also wax esters and glycerides. Peak: 1. Myristic acid, 2. Palmitic acid, 3. Stearic acid, 4. Oleic acid, 5. 18-MEA.

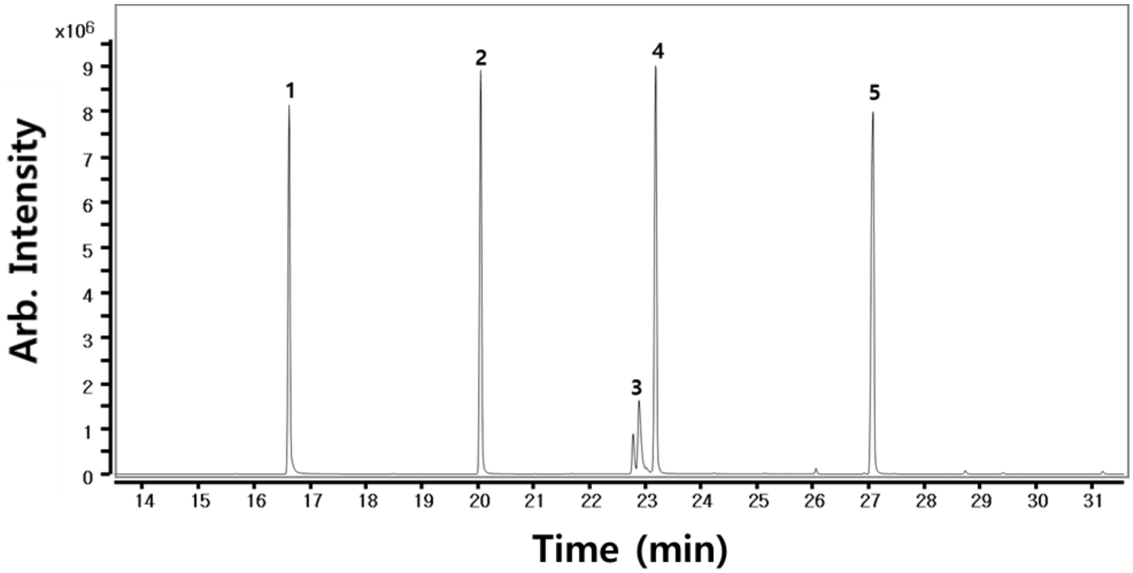

Supplementary Fig. 7. Normalized concentration of lipids. Numbers indicate the average concentration (μg/g hair) of the lipids extracted from virgin hair. The normalized concentrations were calculated by dividing these concentrations of the control. Error bars represent the standard deviation (SD) (n = 6).

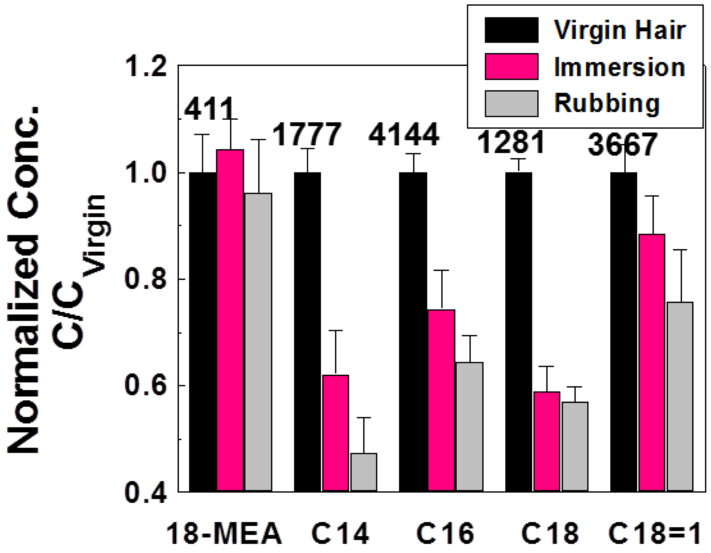

Supplementary Fig. 7 exhibits the total amounts of covalently and noncovalently bound components extracted after methylation. Since the hair was pre-rinsed to remove peripheral lipids (Supplementary Table 4), all lipids found by GC/MS were originally embedded in the hair. The amount of 18-MEA located at the outermost side of the cuticle and covalently bound to underlying proteins through a thioester linkage did not show a significant difference between before and after washing. However, the concentration of fatty acids decreased compared to that of the virgin hair. These findings mean that cuticles layers were not peeled off and lipids were lost from the inside of the hair. Since these fatty acids include those derived from wax esters and triglycerides, it is difficult to characterize the individual behavior of lipids in response to surfactants. Therefore, further experiments (Fig. 1b) were carried out for noncovalently bound components.

Supplementary Fig. 8. SEM images of samples embedded in paraffin (A), epoxy (B), and conventional adhesive tape (C).

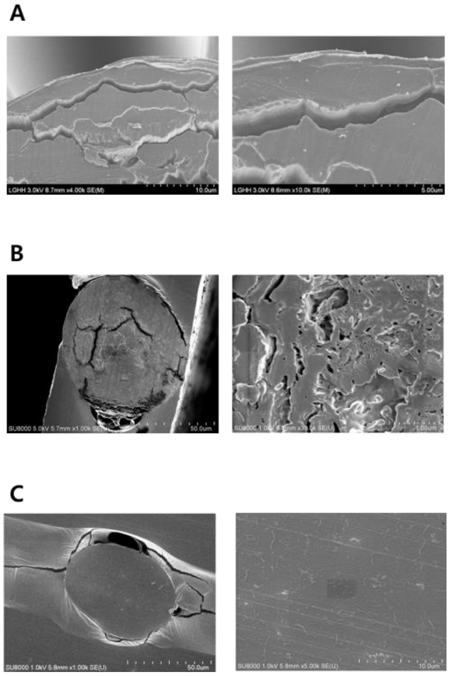

Quantitative data acquisition for spectral mapping demands cross-sectioned hair. Since the conventional methods for sectioning involve a process of removing the embedding material by solvent, the solvent may definitely induce lipid loss from hair. A better method is to perform simple infiltration, which does not require the removal of embedding material.

We tried to embed hair in paraffin (A), epoxy resin (B) and conventional adhesive tape (3M Scotch Cat. 810D) (C) without removing the embedding material. After a rubbing wash, hair was embedded in paraffin or epoxy resin (Struers, Denmark) to allow either longitudinal or transversal sectioning. The isolated hair was subjected to an ultramicrotome to obtain a flattened preparation.

To obtain a consistent focus in each spot, the sectioned surface had to be flat to generate correct Raman mapping. The flatness of cross-sectioned hair was confirmed by monitoring scanning electron microscope (SEM) images. The method of using adhesive tape was the best because internal cracks were detected for the other methods, as shown in Supplementary Fig. S8(A) and (B). Finally, the hair was isolated between conventional adhesive tape, as shown in Supplementary Fig. S8(C).

Supplementary Fig. 9. Chemical scheme of a cationic cellulose polymer. α and β are either ethylene oxide or the OH group.

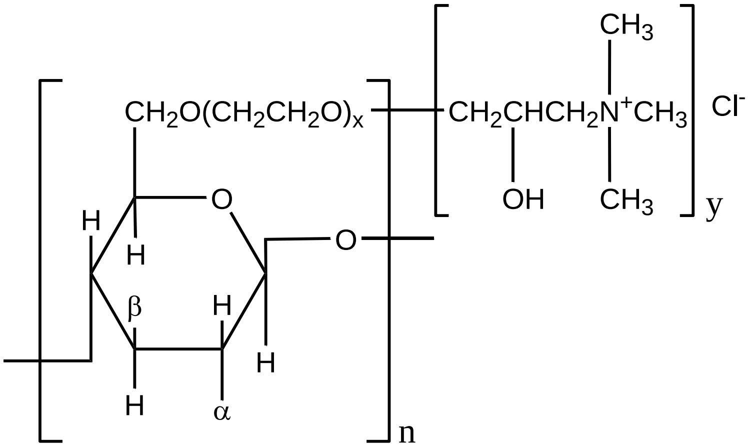

Similar materials are also available from the Dow Chemical Company (US) under the tradename Polymer LR30M for 1.0% (nitrogen density), 3000KC for 2.0%. The polymer with 2.4% nitrogen density is available only from KCI (Korea).

Supplementary Fig. 10. Raman spectra of hair soaked in pure water, in the solution of 5% polylysine, and in the solution of 5% polylysine and 5% PCI. The Raman spectra of the up and bottom panel, which indicates from different hair swatch, are similar. The spectral intensity in cuticle is higher than that in cortex. Intensity of the amide III changes only after internal modification.

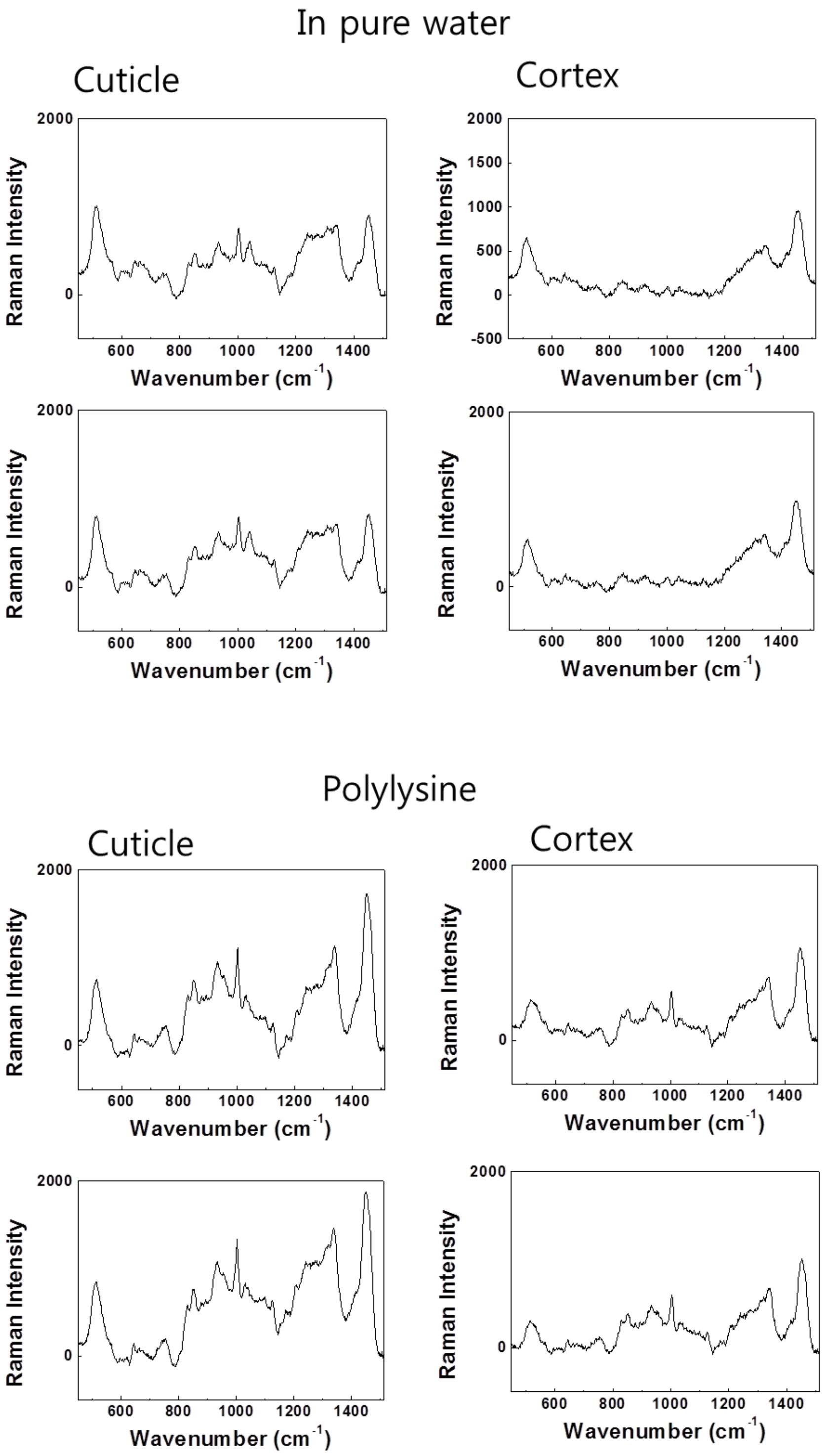

Polylysine and PCI

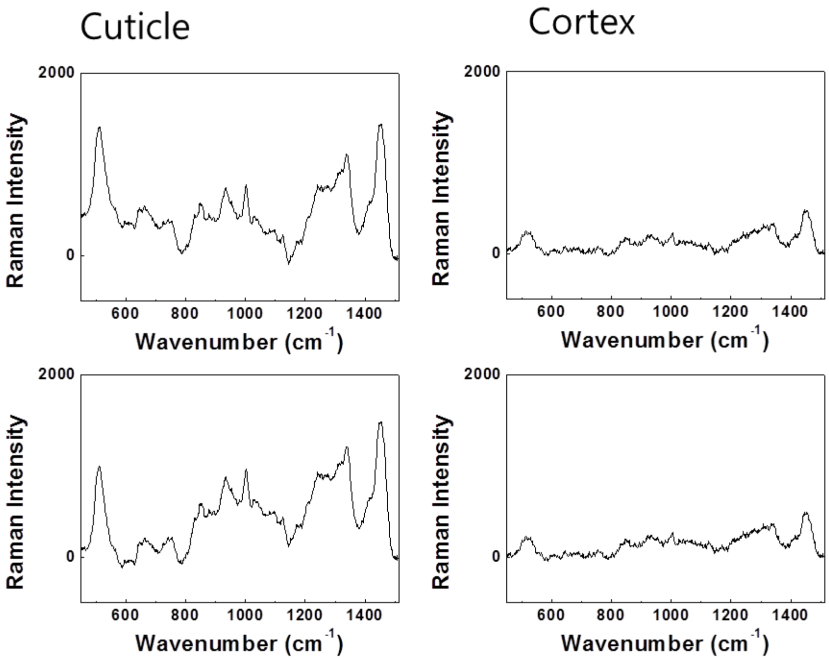

Tape for embedding

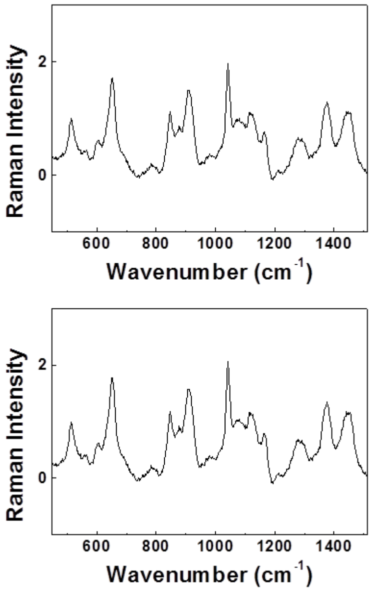

Supplementary Fig. 11. Concentration of lipids after immersion washing and rubbing washing. The x-axis in A and B indicates the period when hair contacted the surfactant. The duration of the washing cycles for the rubbing wash equals the time for immersion washing (1 cycle = 1 min). C16 (A) and C16-C16 (B) are shown as one of the lipids in groups A and B, respectively. In (A), the difference between immersion and rubbing washing increased with the number of washes. In (B), the difference between immersion and rubbing wash was maximized at 10 washing times. The patterns of C14, C18, C18=1, and cholesterol were same as those in (A), and the patterns of squalene, C14-C16, and C18-C16 were same as those in (B).

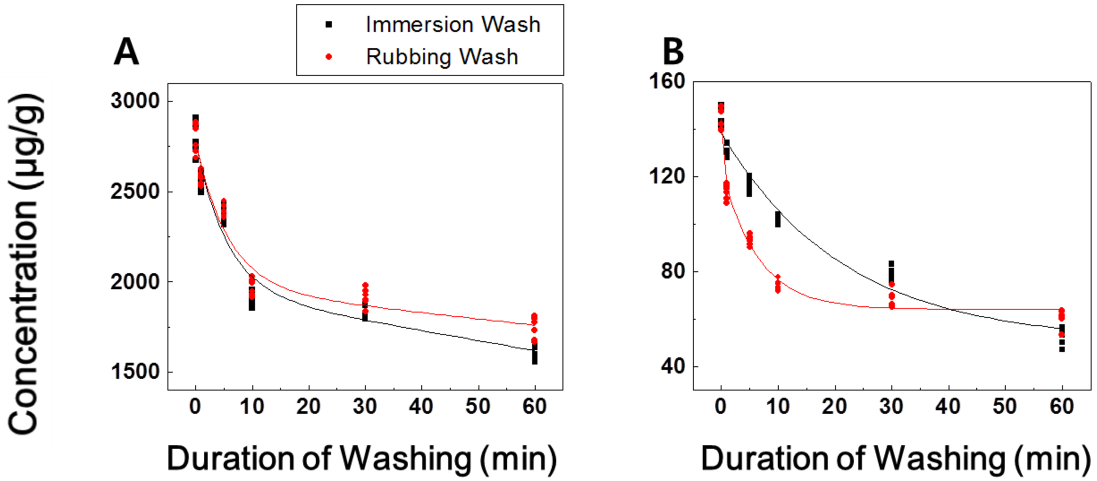

Supplementary Fig. 12. Micellar size analysis. The analysis indicates that the size distribution by intensity has a peak, recorded as 1.45 nm.

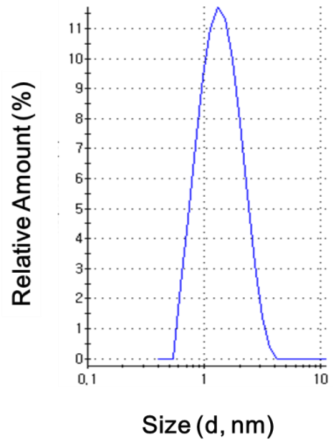

Supplementary Fig. 13. Internal modification with APTES ((3-aminopropyl)triethoxysilane). Hair was rubbing-washed 10 times with a 10% solution of SLES. Control: concentration of virgin hair rubbing-washed 10 times. The results in the graph were normalized to the concentration of the control. The number indicates the average concentration from 6 samples. The error bar indicates the SD. Graphs for the lipids in group A: 1. Myristic acid; 2. Palmitic acid; 3. Stearic acid; 4. Oleic acid; 5. Cholesterol. Graphs for the lipids in group B: 6. Squalene; 7. Myristyl palmitate; 8. Palmityl palmitate; 9. Stearyl palmitate.

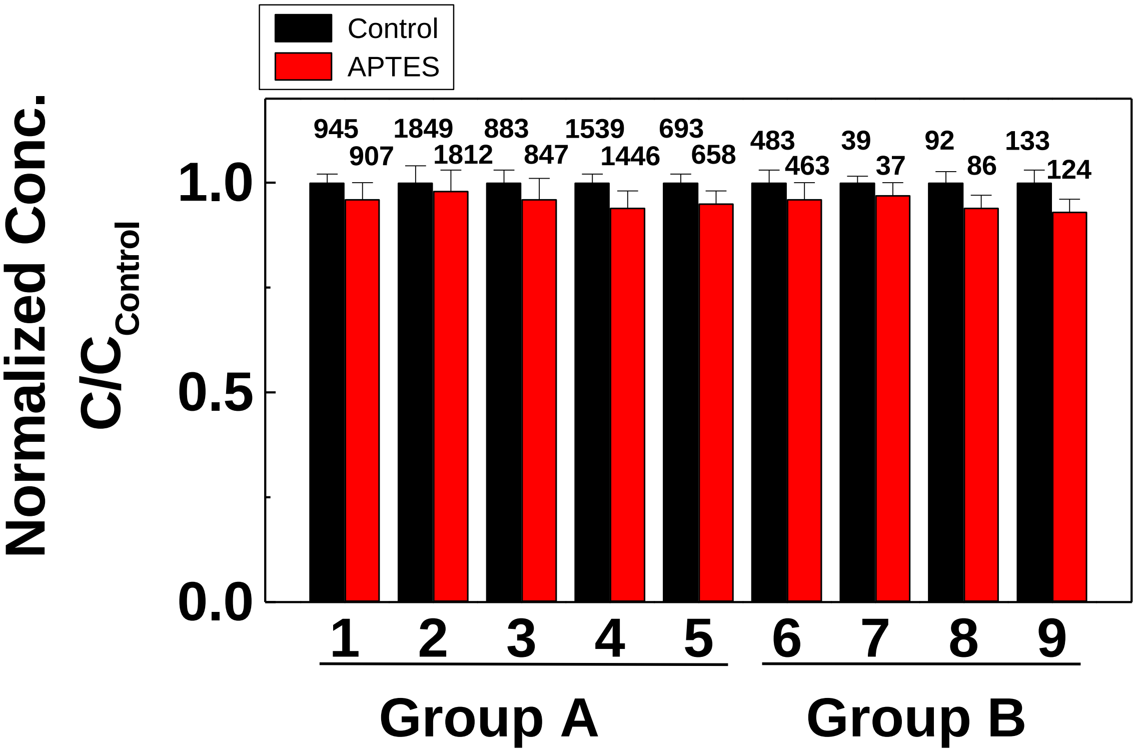

References

1. Kim, J. H.; Park, C. W.; Um, D.; Baek, K. H.; Jo, Y.; An, H.; Kim, Y.; Kim, T. J., Mass spectrometric screening of ovarian cancer with serum glycans. *Dis Markers* **2014,** *2014*, 634289. DOI: 10.1155/2014/634289.
2. Robbins, C. R., *Chemical and Physical Behavior of Human Hair*. 5th ed.; Springer: Heidelberg, 2012. DOI: 10.1007/978-3-642-25611-0.
3. Fujii, T., Hair keratin film as a substitute device for human hair. *Journal of Biological Macromolecules* **2012,** *12* (1), 3-15. DOI: 10.14533/jbm.12.3.
4. Pelipenko, J.; Kristl, J.; Rosic, R.; Baumgartner, S.; Kocbek, P., Interfacial rheology: an overview of measuring techniques and its role in dispersions and electrospinning. *Acta Pharm* **2012,** *62* (2), 123-40. DOI: 10.2478/v10007-012-0018-x.
5. Agency, U. E. P. CAMEO Chemicals version 2.7 rev 3. Chemicals.
6. Masukawa, Y.; Narita, H.; Imokawa, G., Characterization of the lipid composition at the proximal root regions of human hair *J. Cosmet Sci.* **2005,** *56*, 1-16.
7. Wu, Y.; Chen, G.; Ji, C.; Hoptroff, M.; Jones, A.; Collins, L. Z.; Janssen, H. G., Gas chromatography-mass spectrometry and Raman imaging measurement of squalene content and distribution in human hair. *Anal. Bioanal. Chem.* **2016,** *408* (9), 2357-62. DOI: 10.1007/s00216-016-9335-0.
8. Duvel, L.; Chun, H.; Deppa, D.; Wertz, P. W., Analysis of hair lipids and tensile properties as a function of distance from scalp. *Int. J. Cosmetic Sci.* **2005,** *27*, 193–197.
9. Takahashi, T.; Yoshida, S., Distribution of glycolipid and unsaturated fatty acids in human hair. *Lipids* **2014,** *49* (9), 905-17. DOI: 10.1007/s11745-014-3937-0.
10. Auwarter, V.; Kiebling, B.; Pragst, F., Squalene in hair – a natural reference substance for the improved interpretation of fatty acid ethyl ester concentrations with respect to alcohol misuse. *Forensic Sci. Int.* **2004,** *145*, 149-159.
11. Ntsefong, G. N.; Mgando, G.; Ualen, M.; A., N.-M., GC-FID method development and validation parameters for analysis of palm oil (elaeis guineensis jacq.). Fatty acids composition. *Res. Plant Sci.* **2014,** *2* (3), 53-66.
12. Masukawa, Y.; Tsujimura, H.; Imokawa, G., A systematic method for the sensitive and specific determination of hair lipids in combination with chromatography. *Journal of Chromatography B* **2005,** *823* (2), 131-142. DOI: 10.1016/j.jchromb.2005.06.014.
13. Koch, J.; Aitzetmüller, K.; Bittorf, G.; Waibel, J., Hair lipids and their contribution to the perception of hair oiliness: Part I: Surface and internal lipids in hair. *J. Soc. Cosmet. Chem.* **1982,** *33*, 317-326.
14. Rygula, A.; Majzner, K.; Marzec, K. M.; Kaczor, A.; Pilarczyka, M.; Baranskaa, M., Raman spectroscopy of proteins: a review. *J. Raman Spectrosc.* **2013,** *44*, 1061-1076.
15. Natarajan, U.; Robbins, C. R., The thickness of 18-MEA on an ultra-high sulfur protein surface by molecular modeling. *J. Cosmet. Sci.* **2010,** *61* (6), 467-477.
16. Masukawa, Y.; Tsujimura, H.; Narita, H., Liquid chromatography-mass spectrometry for comprehensive profiling of ceramide molecules in human hair. *J. Lipid Res.* **2006,** *47* (7), 1559-71. DOI: 10.1194/jlr.D600007-JLR200.
17. Goldschmidt, D.; Kligman, A., Increased sebum secretion following selenium sulfide shampoos. *Acta Derm. Neurol.* **1968,** *48*, 489-491.
